# A Hierarchical Kinetic Theory of Birth, Death and Fission in Age-Structured Interacting Populations

**DOI:** 10.1007/s10955-016-1524-x

**Published:** 2016-05-14

**Authors:** Tom Chou, Chris D. Greenman

**Affiliations:** Departments of Biomathematics and Mathematics, UCLA, Los Angeles, CA 90095-1766 USA; School of Computing Sciences, University of East Anglia, Norwich, NR4 7TJ UK

**Keywords:** Age structure, Birth-death process, Kinetics, Fission

## Abstract

We develop mathematical models describing the evolution of stochastic age-structured populations. After reviewing existing approaches, we formulate a complete kinetic framework for age-structured interacting populations undergoing birth, death and fission processes in spatially dependent environments. We define the full probability density for the population-size age chart and find results under specific conditions. Connections with more classical models are also explicitly derived. In particular, we show that factorial moments for non-interacting processes are described by a natural generalization of the McKendrick-von Foerster equation, which describes mean-field deterministic behavior. Our approach utilizes mixed-type, multidimensional probability distributions similar to those employed in the study of gas kinetics and with terms that satisfy BBGKY-like equation hierarchies.

## Introduction

Ageing is an important controlling factor in populations of organisms ranging in size from single cells to multicellular animals. Age-dependent population dynamics, where birth and death rates depend on an organism’s age, are important in quantitative models of demography [29], biofilm formation [3], stem cell differentiation [40, 44], and lymphocyte proliferation and death [52]. For example, cellular replication is controlled by a cycle [36, 38, 50], while higher organisms give birth depending on their maturation time. For applications involving small numbers of individuals, a stochastic description of the age-structured population is also desirable. A practical mathematical framework that captures age structure, intrinsic stochasticity, and interactions in a population would be useful for modeling many applications.

Standard frameworks for analyzing age-structured populations include Leslie matrix models [6, 31, 32], which discretizes ages into discrete bins, and the continuous-age McKendrick-von Foerster equation, first studied by McKendrick [28, 34] and subsequently von Foerster [47], Gurtin and MacCamy [17, 18], and others [24, 49]. These approaches describe deterministic dynamics; stochastic fluctuations in population size are not incorporated. On the other hand, intrinsic stochasticity and fluctuations in total population are naturally studied via the Kolmogorov master equation [7, 27]. However, the structure of the master equation implicitly assumes exponentially distributed event (birth and death) times, precluding it from being used to describe age-dependent rates or age structure within the population. Evolution of the generating function associated with the probability distribution for the entire population has also been studied [4, 8, 39, 41]. While this approach, the Bellman-Harris equation, allows for age-dependent event rates, an assumption of independence precludes population-dependent event rates. More recent methods [19, 22, 23, 26] have utilized Martingale approaches, which have been used mainly to investigate the asymptotics of age structure, coalescents, and estimation of Malthusian growth rates.

What is currently lacking is a complete mathematical framework that can resolve the age structure of a population at all time points, incorporate stochastic fluctuations, and be straightforwardly adapted to treat nonlinear interactions such as those arising in populations constrained by a carrying capacity [45, 46]. In a recent publication [16], we took a first step in this direction by formulating a full kinetic equation description that captures the stochastic evolution of the entire age-structured population and interactions between individuals. Here, we generalize the kinetic equation approach introduced in [16] along two main directions. First, we quantify the corrections to the mean-field equations by showing that the factorial moments of the stochastic fluctuations follow an elegant generalization of the McKendrick-von Foerster equation. Second, we show how the methods in [16] can be extended to incorporate fission processes, where single individuals instantaneously split into two identical zero-age offspring. These methods are highlighted with cell division and spatial models.

In the next section, we give a detailed overview of the different techniques currently employed in age-structured population modeling. In Sect. 3, we use previous results [16] to show how the moments of age-structured population size obey a generalized McKendrick-von Foerster equation. In Sect. 4, we expand the kinetic theory for branching processes involving fission. In Sect. 5, we demonstrate how our theory of fission can be applied to a microscopic model of cell growth. In Sect. 6, we demonstrate how to incorporate spatial effects. Conclusions complete the paper.

## Age-Structured Population Modelling

Here we review, compare, and contrast existing techniques of population modeling: the McKendrick-von Foerster equation, the master equation, the Bellman-Harris equation, Leslie matrices, Martingale methods, and our recently introduced kinetic approach [16].

### McKendrick-von Foerster Equation

It is instructive to first outline the basic structure of the classical McKendrick-von Foerster deterministic model as it provides a background for a more complete stochastic picture. First, one defines $\rho (a,t)$ such that $\rho (a,t)\mathrm{d}a$ is the expected number of individuals with age within the interval $[a,a+\mathrm{d}a]$. The total number of organisms at time *t* is thus $n(t) = \int _{0}^{\infty } \rho (a,t)\mathrm{d}a$. Suppose each individual has a rate of giving birth $\beta (a)$ that is a function of its age *a*. For example, $\beta (a)$ may be a function peaked around the time of M phase in a cell cycle or around the most fecund period of an organism. Similarly, $\mu (a)$ is an organism’s rate of dying, which typically increases with its age *a*.

The McKendrick-von Foerster equation is most straightforwardly derived by considering the total number of individuals with age in [0, *a*]: $N(a,t) = \int _{0}^{a}\rho (y,t)\mathrm{d}y$. The number of births per unit time from all individuals into the population of individuals with age in [0, *a*] is $B(t) = \int _{0}^{\infty } \beta (y)\rho (y,t)\mathrm{d}y$, whilst the number of deaths per unit time within this cohort is $D(a,t) =\int _{0}^{a}\mu (y)\rho (y,t)\mathrm{d}y$. Within a small time window $\varepsilon$, the change in *N*(*a*, *t*) is

1 $$\begin{aligned} N(a+\varepsilon ,t+\varepsilon ) - N(a,t) = \int _{t}^{t+\varepsilon }B(s)\mathrm{d}s - \int _{0}^{\varepsilon }D(a+s,t+s)\mathrm{d}s. \end{aligned}$$

In the $\varepsilon \rightarrow 0$ limit, we find

2 $$\begin{aligned} {\partial N(a,t)\over \partial t} + {\partial N(a,t)\over \partial a}&= \int _{0}^{a} \dot{\rho }(y,t)\mathrm{d}y + \rho (a,t) = B(t) -\int _{0}^{a} \mu (y)\rho (y,t)\mathrm{d}y. \end{aligned}$$

Upon taking ${\partial \over \partial a}$ of Eq. 2, we obtain the McKendrick-von Foerster equation:

3 $$\begin{aligned} {\partial \rho (a,t)\over \partial t} + {\partial \rho (a,t)\over \partial a} = -\mu (a)\rho (a,t). \end{aligned}$$

The associated boundary condition arises from setting $a=0$ in Eq. 2:

4 $$\begin{aligned} \rho (a=0,t) = \int _{0}^{\infty } \beta (y)\rho (y,t)\mathrm{d}y \equiv B(t). \end{aligned}$$

Finally, an initial condition $\rho (a,t=0) = g(a)$ completely specifies the mathematical model.

Note that the term on the right-hand side of Eq. 3 depends only on death; the birth rate arises in the boundary condition (Eq. 4) since births give rise to age-zero individuals. These equations can be formally solved using the method of characteristics. The solution to Eqs. 3 and 4 that satisfies a given initial condition is5To explicitly identify the solution, we need to calculate the fecundity function *B*(*t*). By substituting Eq. 5 into the boundary condition of Eq. 4 and defining the propagator $U(a_1,a_2)\equiv \exp \left[ -\int _{a_1}^{a_2}\mu (s)\mathrm{d}s \right]$, we obtain the following Volterra integral equation:

6 $$\begin{aligned} B(t) = \int _0^t B(t-a)U(0,a)\beta (a) \mathrm{d}a + \int _0^\infty g(a)U(a,a+t)\beta (a+t) \mathrm{d}a. \end{aligned}$$

After Laplace-transforming with respect to time, we find

7 $$\begin{aligned} \hat{B}(s) = \hat{B}(s)\mathcal {L}_s\left\{ U(0,t)\beta (t)\right\} +\int _0^\infty g(a)\mathcal {L}_s\left\{ U(a,a+t)\beta (a+t)\right\} \mathrm{d}a. \end{aligned}$$

Solving the above for $\hat{B}(s)$ and inverse Laplace-transforming, we find the explicit expression

8 $$\begin{aligned} B(t) = \mathcal {L}_t^{-1}\left\{ \frac{\int _0^\infty g(a)\mathcal {L}_s\left\{ U(a,a+t)\beta (a+t)\right\} \mathrm{d}a}{1-\mathcal {L}_s\left\{ U(0,t)\beta (t)\right\} } \right\} , \end{aligned}$$

which provides the complete solution when used in Eq. 5.

The McKendrick-von Foerster equation is a deterministic model describing only the expected age distribution of the population. If one integrates Eq. 3 across all ages $0\le a < \infty$ and uses the boundary conditions, the rate equation for the total population is $\dot{n}(t) = \int _{0}^{\infty }(\beta (a)-\mu (a))\rho (a,t)\mathrm{d}a$. Generally, *n*(*t*) will diverge or vanish in time depending on the details of $\beta (a)$ and $\mu (a)$. In the special case $\beta (a) = \mu (a)$, the population is constant.

What is missing are interactions that stabilize the total population. Eqs. 3 and 4 assume no higher-order interactions (such as competition for resources, a carrying capacity, or mating patterns involving pairs of individuals) within the populations. Within the McKendrick-von Foerster theory, interactions are typically incorporated via population-dependent birth and death rates, $\beta (a;n(t))$ and $\mu (a;n(t))$, respectively [11, 17, 18]. The McKendrick-von Foerster equation must then be self-consistently solved. However, as shown in [16], this assumption is an uncontrolled approximation and inconsistent with a detailed microscopic stochastic model of birth and death.

### Master Equation Approach

A popular way to describe stochastic birth-death processes is through a function $\rho _{n}(t)$ defining the probability that a population contains *n* identical individuals at time *t*. The evolution of this process can then be described by the standard forward continuous-time master equation [7, 27]

9 $$\begin{aligned} {\partial \rho _{n}(t)\over \partial t} = -n\left[ \beta _n(t)+\mu _n(t)\right] \rho _n(t) +(n-1)\beta _{n-1}(t)\rho _{n-1}(t) +(n+1)\mu _{n+1}(t)\rho _{n+1}(t),\nonumber \\ \end{aligned}$$

where $\beta _{n}(t)$ and $\mu _{n}(t)$ are the birth and death rates, per individual, respectively. Each of these rates can be population-size- and time-dependent. As such, Eq. 9 explicitly includes the effects of interactions. For example, a carrying capacity can be implemented into the birth rate through the following form:

10 $$\begin{aligned} \beta _{n}(t) = \beta _{0}(t)\left( 1-{n\over K(t)}\right) . \end{aligned}$$

Here, we have allowed both the intrinsic birth rate $\beta _{0}(t)$ and the carrying capacity *K*(*t*) to be functions of time. Eq. 9 can be analytically or numerically solved via generating function approaches, especially for simple functions $\beta _{n}$ and $\mu _{n}$.

Since $\rho _{n}(t)$ only describes the total number of individuals at time *t*, it cannot resolve the distribution of ages within the fluctuating population. Another shortcoming is the implicit assumption of exponentially distributed waiting times between birth and death events. The times since birth of individuals are not tracked. General waiting time distributions can be incorporated into a master equation approach by assuming an appropriate number of internal “hidden” states, such as the different phases in a cell division cycle [50]. After all internal states have been sequentially visited, the system makes a change to the external population-size state. The waiting time between population-size changes is then a multiple convolution of the exponential waiting-time distributions for transitions along each set of internal states. The resultant convolution can then be used to approximate an arbitrary waiting-time distribution for the effective transitions between external states. It is not clear, however, how to use such an approach to resolve the age structure of the population.

**Fig. 1:**
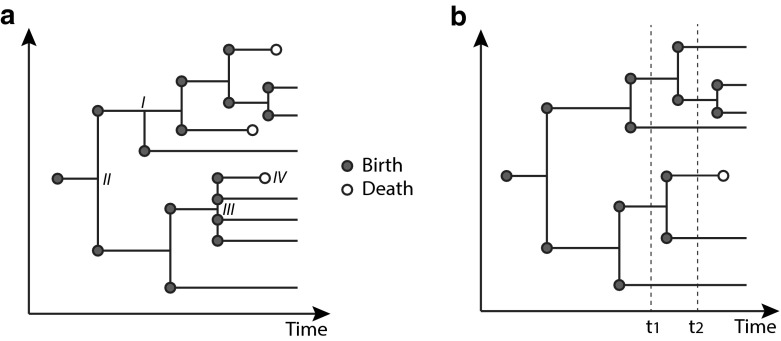
**a** A general branching process. *I* indicates a *budding* or *simple* birth process, where the parental individual produces a single offspring (a ‘singlet’) without death. *II* indicates *binary fission*, where a parent dies at the same moment two newborn *twins* occur (a ‘doublet’). *III* indicates a more general fission event with four offspring (a ‘quadruplet’). *IV* indicates death, which can be viewed as fission with zero offspring. **b** A binary fission process such as cell division. At time $t_1$ we have four individuals; two sets of twins. At time $t_2$ we have six individuals; two pairs of twins and two singlets

### Bellman-Harris Fission Process

The Bellman-Harris process [4, 8, 25, 39, 41] describes fission of a particle into any number of identical daughters, such as events $\mathsf {II}$, $\mathsf {III}$, and $\mathsf {IV}$ in Fig. 1a. Unlike the master equation approach, the Bellman-Harris branching process approach allows interfission times to be arbitrarily distributed. However, it does not model the budding mode of birth indicated by process $\mathsf {I}$ in Fig. 1a, nor does it capture interactions (such as carrying capacity effects) within the population. In such a noninteracting limit, the Bellman-Harris fission process is most easily analyzed using the generating function *F*(*z*, *t*) associated with the probability $\rho _{n}(t)$, defined as

11 $$\begin{aligned} F(z,t) \equiv \sum _{n=0}^{\infty } \rho _{n}(t) z^{n}. \end{aligned}$$

We assume an initial condition consisting of a single, newly born parent particle, $\rho _{n}(0) = \delta _{n,1}$. If we also assume the first fission or death event occurs at time $\tau$, we can define $F(z,t\vert \tau )$ as the generating function conditioned on the first fission or death occurring at time $\tau$ and write *F* recursively [1, 2, 20] as:

12 $$\begin{aligned} F(z,t\vert \tau ) = {\left\{ \begin{array}{ll} z, &{} t<\tau , \\ H(F(z,t-\tau )), &{} t \ge \tau , \end{array}\right. }\quad H(x) = \sum _{m=0}^{\infty } h_{m} x^{m}. \end{aligned}$$

The function *H* encapsulates the probability $h_{m}$ that a particle splits into *m* identical particles upon fission, for each non-negative integer *m*. For binary fission, we have $H(x) = (1-h_{2}) + h_{2}x^{2}$ since $\sum _{m=0}^{\infty }h_{m} = 1$. Since this overall process is semi-Markov [48], each daughter behaves as a new parent that issues its own progeny in a manner statistically equivalent to and independent from the original parent, giving rise to the compositional form in Eq. 12. We now weight $F(z,t\vert \tau )$ over a general distribution of waiting times between splitting events, $g(\tau )$, to find

13 $$\begin{aligned} F(z,t)&\displaystyle \equiv \int _{0}^{\infty }F(z,t \vert \tau )g(\tau )\mathrm{d}\tau \nonumber \\&= z \int _{t}^{\infty }\!\!\!g(\tau )\mathrm{d}\tau + \int _{0}^{t}\!H(F(z,t-\tau ))g(\tau )\mathrm{d}\tau . \end{aligned}$$

The Bellman-Harris branching process [2, 14] is thus defined by two parameter functions: $h_{m}$, the vector of progeny number probabilities, and $g(\tau )$, the probability density function for waiting times between branching events for each particle. The probabilities $\rho _n(t)$ can be recovered using a contour integral (or Taylor expanding) about the origin:

14 $$\begin{aligned} \rho _{n}(t) =\frac{1}{2\pi i}\oint _{C}\frac{F(z,t)}{z^{n+1}} \mathrm{d}z = {1\over n!}{\partial ^{n} F(z,t)\over \partial z^{n}} \bigg |_{z=0}. \end{aligned}$$

Note that Eq. 13 incorporates an arbitrary waiting-time distribution between events, a feature that is difficult to implement in the master equation (Eq. 9). An advantage of the branching process approach is the ease with which general waiting-time distributions, multiple species, and immigration can be incorporated. However, it is limited in that an independent particle assumption was used to derive Eq. 13, where the statistical properties of the entire process starting from one parent were assumed to be equivalent to those started by each of the identical daughters born at time $\tau$. This assumption of independence precludes treatment of interactions within the population, such as those giving rise to carrying capacity. More importantly, the Bellman-Harris equation is expressed purely in terms of the generating function for the total population size and cannot resolve age structure within the population.

### Leslie Matrices

Leslie matrices [31, 32] have been used to resolve the age structure in population models [9, 10, 12, 15, 31–33, 39, 44]. These methods essentially divide age into discrete bins and are implemented by assuming fixed birth and death rates within each age bin. Such approaches have been applied to models of stochastic harvesting [10, 15] and fluctuating environments [13, 30] and are based on the following linear construction, iterated over a single time step:

15 $$\begin{aligned} \begin{bmatrix} n_0\\n_1\\ \vdots \\ n_{N-1} \end{bmatrix}_{t+1}= \begin{bmatrix} f_0&f_1&\cdots&f_{N-2}&f_{N-1} \\ s_0&0&\cdots&0&0 \\ 0&s_1&\cdots&0&0 \\ \vdots&\vdots&\ddots&\vdots&\vdots \\ 0&0&\cdots&s_{N-2}&0 \end{bmatrix} \cdot \begin{bmatrix} n_0\\n_1\\ \vdots \\ n_{N-1} \end{bmatrix}_{t}. \end{aligned}$$

The value $n_i$ indicates the population size in age group *i*; $f_i$ is the mean number of offspring arriving to age group 0 from a parent in age group *i*; and $s_i$ is the fraction of individuals surviving from age group *i* to $i+1$. These models have the advantage of being based upon algebraic linearity, which enables many features of interest to be investigated analytically [6]. However, they are inherently deterministic (although they can be used to study extrinsic environmental noise) and the discretization within such models results in an approximation. Thus, a fully continuous stochastic model is desirable.

### Martingale Approaches

Relatively recent investigations have used Martingale approaches to model age-structured stochastic processes. These methods stem from stochastic differential equations and Dynkin’s formula [37] and evolves general processes of the form $F(f(\mathbf{a}_n(t)))$. Here the vector $\mathbf{a}_n(t)$ represents the time dependent age-chart of the population with variable size *n*; *f* is a symmetric function of the individual ages; and *F* is a generic function of interest. A Martingale decomposition of the following form results

16 $$\begin{aligned} F(f(\mathbf{a}_n(t))) = F(f(\mathbf{a}_n(0))) + \int _0^t \mathcal{G} F(f(\mathbf{a}_n;s)) \mathrm{d}s + M_t^{(f,F)}, \end{aligned}$$

where the operator $\mathcal{G}$ captures the mean behavior, and the stochastic behavior is encoded in the local Martingale process $M_t^{(f,F)}$ [26]. Such analyses have enabled several features of general birth-death processes, including both budding and fission forms of birth to be quantified. Specifically, the Malthusian growth parameter can be explicitly determined, along with the asymptotic behavior of the age structure. More recently there have been results related to coalescents and extinction of these processes [19, 22, 23]. However, we will show the utility of obtaining the probability density of the entire age chart of the population which allows efficient computations in transient regimes. The kinetic approach first developed in [16] introduces machinery to accomplish this.

### Kinetic Theory

A brief introduction to the current formulation of our kinetic theory approach to age-structured populations can be found in [16]. The starting point is a derivation of a variable-dimension coupled set of partial differential equations for the complete probability density function $\rho _n(\mathbf{a}_n;t)$ describing a stochastic, interacting, age-structured population subject to simple birth and death. Variables in the theory include the population size *n*, time *t*, and the vector $\mathbf{a}_n=(a_1,a_2,\ldots ,a_n)$ representing the complete age-chart for the *n* individuals. If we randomly label the individuals $1,2,\ldots ,n$, then $\rho _n(\mathbf{a}_n;t)\mathrm{d}\mathbf{a}_n$ represents the probability that the $i^\mathrm {th}$ individual has age in the interval $[a_i,a_i+\mathrm{d}a_i]$. Since individuals are considered indistinguishable, $\rho _n(\mathbf{a}_n;t)$ is invariant under any permutation of the age-chart vector $\mathbf{a}_{n}$. These functions are analogous to those used in kinetic theories of gases [35]. Their analysis in the context of age-structured populations builds on the Boltzmann kinetic theory of Zanette [51] and results in the kinetic equation

17 $$\begin{aligned}&\displaystyle {\partial \rho _{n}(\mathbf{a}_{n};t)\over \partial t} + \sum _{j=1}^{n}{\partial \rho _{n}(\mathbf{a}_{n};t)\over \partial a_{j}} \nonumber \\&\quad = -\rho _{n}(\mathbf{a}_{n};t)\sum _{i=1}^{n} \gamma _{n}(a_{i}) + (n+1)\!\int _{0}^{\infty }\!\!\mu _{n+1}(y) \rho _{n+1}(\mathbf{a}_{n},y;t)\mathrm{d}y, \end{aligned}$$

where $\gamma _n(a) = \beta _n(a) +\mu _n(a)$ and the age variables are separated from the time variable by a semicolon. The associated boundary condition is

18 $$\begin{aligned} \begin{array}{l} n \rho _{n}(\mathbf{a}_{n-1},0;t) = \rho _{n-1}(\mathbf{a}_{n-1};t)\beta _{n-1}(\mathbf{a}_{n-1}). \end{array} \end{aligned}$$

**Table 1:** Advantages and disadvantages of different frameworks for stochastic age-structured populations. ‘Stochastic’ indicates that the model resolves probabilities of configurations of the population

Theory	Stochastic	Age-dependentrates	Age-structuredpopulations	Age-chart resolved	Interactions	Budding	Fission
Verhulst Eq.	$\times$	$\times$	$\times$	$\times$	$\checkmark$	$\times$	$\times$
McKendrick Eq.	$\times$	$\checkmark$	$\checkmark$	$\times$	$\checkmark$	$\checkmark$ ^1^	$\times$
Master Eq.	$\checkmark$	$\times$	$\times$	$\times$	$\checkmark$	$\checkmark$	$\checkmark$
Bellman-Harris	$\checkmark$	$\checkmark$	$\times$	$\times$	$\times$	$\times$	$\checkmark$
Leslie matrices	$\times$	$\checkmark$ ^2^	$\checkmark$	$\times$	$\checkmark$	$\times$	$\times$
Martingale	$\checkmark$	$\checkmark$	$\times$ ^3^	$\times$	$\checkmark$	$\checkmark$	$\checkmark$
Kinetic theory	$\checkmark$	$\checkmark$	$\checkmark$	$\checkmark$	$\checkmark$	$\checkmark$	$\checkmark$ ^4^

‘Age-dependent rates’ indicates whether or not a model takes into account birth, death, or fission rates that depend on an individuals age (time after its birth). ‘Age-structured Populations’ indicates whether or not the theory outputs the age structure of the ensemble population. ‘Age-chart resolved’ indicates whether or not a theory outputs the age distribution of all the individuals in the population. ‘Interactions’ indicates whether or not the approach can incorporate population-dependent dynamics such as that arising from a carrying capacity, or from birth processes involving multiple parents. ‘Budding’ and ‘Fission’ describes the model of birth and indicates whether the parent lives or dies after birth

^1^ Birth and death rates in the McKendrick-von Foerster equation can be made explicit functions of the total populations size, which must be self-consistently solved [17, 18]

^2^ Leslie matrices discretize age groups and are an approximate method

^3^ Martingale methods do not resolve the age structure explicitly, but utilize rigorous machinery

^4^ The kinetic approach for fission is addressed later in this work, but not in [16]

Note that because $\rho _n(\mathbf{a}_{n-1},0;t)$ is symmetric in the age arguments, the zero can be placed equivalently in any of the *n* age coordinates. The birth rate function can be quite general and can take forms such as $\beta _{n-1}(\mathbf{a}_{n-1})= \sum _{i=1}^{n-1}\beta _{n-1}(a_i)$ for a simple birth process or $\sum _{1 \le i < j \le n-1}\beta _{n-1}(a_i,a_j)$ to represent births arising from interactions between pairs of individuals.

Equation 17 applies only to the budding or simple mode of birth such as event $\mathsf {I}$ in Fig. 1a. In [16] we derived analytic solutions for $\rho _{n}(\mathbf{a}_{n};t)$ in pure death and pure birth processes, and showed that marginal densities obeyed a BBGKY-like (Bogoliubov-Born-Green-Kirkwood-Yvon) hierarchy of equations. Furthermore, when the birth and death rates are age-independent (but possibly number-dependent), the hierarchy of equations reduce to a single master equation for the total number of individuals *n* in the population. Characterizing all the remaining higher moments of the distribution remains an outstanding problem. Moreover, methods to tackle fission modes of birth such as those shown in Fig. 1b were not developed. These are the two contributions described in this paper. Before analyzing these problems, we summarize the pros and cons of the different approaches in Table 1.

## Analysis of Simple Birth-Death Processes

We now revisit the simple process of budding birth and death, and extend the kinetic framework introduced in [16]. We first show that the factorial moments for the density $\rho _n(\mathbf{a}_n;t)$ satisfy a generalized McKendrick-von Foerster equation. We also explicitly solve Eqs. 17 and 18, and derive for the first time an exact general solution for $\rho _n(\mathbf{a}_n;t)$.

### Moment Equations

The McKendrick-von Foerster equation has been shown to correspond to a mean-field theory of age-structured populations in which the birth and death rates $\beta (a)$ and $\mu (a)$ are population-independent [16]. This leaves open the problem of determining the age-structured variance (and higher-order moments) of the population size.

In [16], we derived the marginal *k*-dimensional distribution functions defined by integrating $\rho _n(\mathbf{a}_n;t)$ over $n-k$ age variables:

19 $$\begin{aligned} \rho _{n}^{(k)}(\mathbf{a}_{k};t) \equiv \int _{0}^{\infty }\!\mathrm{d}a_{k+1} \ldots \int _{0}^{\infty }\!\mathrm{d}a_{n}\, \rho _{n}(\mathbf{a}_{n};t). \end{aligned}$$

The symmetry properties of $\rho _{n}(\mathbf{a}_{n};t)$ indicate that it is immaterial which of the $n-k$ age variables are integrated out. From Eq. 17, we then obtained

20 $$\begin{aligned} \displaystyle {\partial \rho _{n}^{(k)}(\mathbf{a}_{k};t) \over \partial t} + \sum _{i=1}^{k}{\partial \rho _{n}^{(k)} (\mathbf{a}_{k};t)\over \partial a_{i}} =&\displaystyle - \rho _{n}^{(k)}(\mathbf{a}_{k};t)\sum _{i=1}^{k}\gamma _{n}(a_{i}) \nonumber \\ \,&+ \left( {n-k \over n}\right) \rho _{n-1}^{(k)}(\mathbf{a}_{k};t) \sum _{i=1}^{k}\beta _{n-1}(a_{i}) \nonumber \\ \,&+{(n-k)(n-k-1)\over n}\int _{0}^{\infty }\beta _{n-1}(y) \rho _{n-1}^{(k+1)}(\mathbf{a}_{k},y;t)\mathrm{d}y \nonumber \\ \,&-(n-k)\int _{0}^{\infty }\gamma _{n}(y) \rho _{n}^{(k+1)}(\mathbf{a}_{k},y;t)\mathrm{d}y \\ \,&\displaystyle + (n+1)\int _{0}^{\infty }\mu _{n+1}(y) \rho _{n+1}^{(k+1)}(\mathbf{a}_{k},y;t)\mathrm{d}y.\nonumber \end{aligned}$$

Similarly, integrating the boundary condition in Eq. 18 over $n-k$ of the (nonzero) variables, gives, for simple birth processes where $\beta _n(\mathbf{a}_m)= \sum _{i=1}^m \beta _n(a_i)$,

21 $$\begin{aligned} \rho _n^{(k)}(\mathbf{a}_{k-1},0;t) = \frac{1}{n}\rho _{n-1}^{(k-1)}(\mathbf{a}_{k-1};t)\sum _{i=1}^{k-1} \beta _{n-1}(a_i)+\frac{n-k}{n}\int _0^\infty \rho _{n-1}^{(k)}(\mathbf{a}_{k-1},y;t)\beta _{n-1}(y)\mathrm{d}y. \end{aligned}$$

We now show how to use these marginal density equation hierarchies and boundary conditions to derive an equation for the $k^\mathrm {th}$ moment of the age density.

For $k=1$, $\rho _{n}^{(1)}(a;t)\mathrm{d}a$ is the probability that we have *n* individuals and that if one of them is randomly chosen, it will have age in $[a,a+\mathrm{d}a]$. Therefore, the probability that we have *n* individuals, and that there exists an individual with age in $[a,a+\mathrm{d}a]$, is $n\rho _{n}^{(1)}(a; t)\mathrm{d}a$. Summing over all possible population sizes $n\ge 1$ yields the probability $\rho (a,t)\mathrm{d}a = \sum _nn\rho _n^{(1)}(a;t)\mathrm{d}a$ that the system contains an individual with age in the interval $[a,a+\mathrm{d}a]$. More generally, $n^k\rho _n^{(k)}(\mathbf{a}_k;t)\mathrm{d}\mathbf{a}_k$ is the probability that there are *n* individuals, *k* of which can be labelled such that the $i^\mathrm {th}$ has age within the interval $[a_i,a_i+\mathrm{d}a_i]$. Summing over the possibilities $n \ge k$, we thus introduce factorial moments $X^{(k)}(\mathbf{a}_k;t)$ and moment functions $Y^{(k)}(\mathbf{a}_k;t)$ as:

22 $$\begin{aligned} X^{(k)}(\mathbf{a}_k;t) \equiv \sum _{n=k}^{\infty }(n)_k\rho _{n}^{(k)}(\mathbf{a}_k;t) \equiv \sum _{\ell =0}^k s(k,\ell )Y^{(\ell )}(\mathbf{a}_\ell ;t),\nonumber \\ Y^{(k)}(\mathbf{a}_k;t) \equiv \sum _{n=k}^{\infty }{n^k}\rho _{n}^{(k)}(\mathbf{a}_k;t) \equiv \sum _{\ell =0}^k S(k,\ell )X^{(\ell )}(\mathbf{a}_\ell ;t). \end{aligned}$$

Here $(n)_k = n(n-1)\cdots (n-(k-1))=k!{n\atopwithdelims ()k}$ is the Pochhammer symbol, and $s(k,\ell )$ and $S(k,\ell )$ are Stirling numbers of the first and second kind, respectively [42, 43]. Although we are primarily interested in the functions $Y^{(k)}(\mathbf{a}_k;t)$, the factorial moments $X^{(k)}(\mathbf{a}_k;t)$ will prove to be analytically more tractable. One can then easily interchange between the two moment types by using the polynomial relationships involving Stirling numbers.

After multiplying Eq. 20 by $(n)_k$ and summing over all $n \ge k$, we find

23 $$\begin{aligned} \frac{\partial X^{(k)}}{\partial t}+ \sum _{i=1}^{k}\frac{\partial X^{(k)}}{\partial a_i} +\sum _{n \ge k}(n)_k\rho _{n}^{(k)}&\sum _{i=1}^{k}\gamma _{n}(a_{i}) = \sum _{n-1 \ge k}\!(n-1)_k\rho _{n-1}^{(k)} \sum _{i=1}^{k}\beta _{n-1}(a_{i}) \nonumber \\&+\int _{0}^{\infty }\!\!\sum _{n-1 \ge k+1}(n-1)_{k+1}\rho _{n-1}^{(k+1)}(\mathbf{a}_{k},y;t)\beta _{n-1}(y)\mathrm{d}y \nonumber \\&-\int _{0}^{\infty }\!\!\sum _{n \ge k+1}(n)_{k+1}\rho _{n}^{(k+1)}(\mathbf{a}_{k},y;t)\gamma _{n}(y)\mathrm{d}y \nonumber \\&+\int _{0}^{\infty }\!\!\sum _{n+1 \ge k+1}(n+1)_{k+1}\rho _{n+1}^{(k+1)}(\mathbf{a}_{k},y;t)\mu _{n+1}(y)\mathrm{d}y, \end{aligned}$$

where, for simplicity of notation, the arguments $(\mathbf{a}_k;t)$ have been suppressed from $\rho _{n}^{(k)}$ and $X^{(k)}$. In the case where the birth and death rates $\beta _n(a)=\beta (a)$ and $\mu _n(a)=\mu (a)$ are independent of the sample size, significant cancellation occurs and we find the simple equation

24 $$\begin{aligned} \frac{\partial X^{(k)}}{\partial t}+ \sum _{i=1}^{k}\frac{\partial X^{(k)}}{\partial a_i} +X^{(k)}\sum _{i=1}^{k}\mu (a_{i}) = 0. \end{aligned}$$

When $k=1$, one recovers the classical McKendrick-von Foerster equation describing the mean-field behavior after stochastic fluctuations are averaged out. Equation 24 is a natural generalization of the McKendrick-von Foerster equation and provides all the age-structured moments arising from the population size fluctuations. If the birth and death rates, $\beta _{n}$ and $\mu _{n}$, depend on the population size, one has to analyze the complicated hierarchy given in Eq. 23.

To find the boundary conditions associated with Eq. 24, we combine the definition of $X^{(k)}$ with the boundary condition in Eq. 21 and obtain

25 $$\begin{aligned} X^{(k)}(\mathbf{a}_{k-1},0;t) =&\sum _{n \ge k}(n)_k\rho _n^{(k)}(\mathbf{a}_{k-1},0;t)\nonumber \\ =\,&X^{(k-1)}(\mathbf{a}_{k-1};t)\beta (\mathbf{a}_{k-1})+\int _0^\infty X^{(k)}(\mathbf{a}_{k-1},y;t)\beta (y) \mathrm{d}y. \end{aligned}$$

Setting $X^{(0)}\equiv 0$, we recover the boundary condition associated with the classical McKendrick-von Foerster equation. For higher-order factorial moments, the full solution to the $(k-1)^\mathrm {st}$ factorial moment $X^{(k-1)}(\mathbf{a}_{k-1};t)$ is required for the boundary condition to the $k^\mathrm {th}$ moment $X^{(k)}(\mathbf{a}_{k-1},0;t)$.

Specifically, consider the second factorial moments and assume the solution $X^{(1)}\equiv Y^{(1)}$ to the McKendrick-von Foerster equation is available (from e.g., Eq. 5). In the infinitesimal interval $\mathrm{d}a$, the term $Y^{(1)}\mathrm{d}a$ is the Bernoulli variable for an individual having an age in the interval $[a,a+da]$. Thus, in an extended age window $\Omega$, we heuristically obtain the expectation

26 $$\begin{aligned} \mathrm {E}(Y_\Omega (t)) = \sum _{da \in \Omega }Y_{da}(t)=\int _\Omega Y^{(1)}(a;t) \mathrm{d}a, \end{aligned}$$

where $Y_\Omega (t)$ is the stochastic random variable describing the number of individuals with an age in $\Omega$ at time *t*. Using an analogous argument for the variance, we find

27 $$\begin{aligned} \mathrm {Var}(Y_\Omega (t))&=\sum _{da,db \in \Omega } Cov(Y_{da},Y_{db})\nonumber \\&=\int _{\Omega ^2}Y^{(2)}(a,b;t)\mathrm{d}a \mathrm{d}b-\int _\Omega Y^{(1)}(a;t)\mathrm{d}a \cdot \int _\Omega Y^{(1)}(b;t) \mathrm{d}b. \end{aligned}$$

Thus, the second moment $Y^{(2)}$ allows us to describe the variation of the population size within any age region of interest. Similar results apply for higher order correlations. We focus then on deriving a solution to $Y^{(2)}$ and determining the variance of population-size-age-structured random variables. Eq. 24 for general *k* is readily solved using the method of characteristics leading to

28 $$\begin{aligned} X^{(k)}(\mathbf{a}_k;t)=X^{(k)}(\mathbf{a}_k-m;t-m)\prod _{j=1}^kU(a_j-m,a_j), \end{aligned}$$

where the propagator is defined as $U(a,b) \equiv \exp \left[ -\int _a^b \mu (s) \mathrm{d}s\right]$. We can now specify $X^{(k)}$ in terms of boundary conditions and initial conditions by selecting $m = \min \left\{ \mathbf{a}_k,t\right\}$. Since $X^{(k)}(\mathbf{a}_k;t)\equiv X^{(k)}(\pi (\mathbf{a}_k);t)$ is invariant to any permutation $\pi$ of its age arguments, we have only two conditions to consider. The initial condition $X^{(k)}(\mathbf{a}_k;0)=g(\mathbf{a}_k)$ encodes the initial correlations between the ages of the founder individuals and is assumed to be given. From Eq. 22, $X^{(k)}(\mathbf{a}_k;0)$ must be a symmetric function in the age arguments. A boundary condition of the form $X^{(k)}(\mathbf{a}_{k-1},0;t)\equiv B(\mathbf{a}_{k-1};t)$ describes the fecundity of the population through time. This is not given but can be determined in much the same way that Eq. 8 was derived.

To be specific, consider a simple pure birth (Yule-Furry) process ($\beta (a) = \beta$, $\mu (a) = 0$) started by a single individual. The probability distribution of the initial age of the parent individual is assumed to be exponentially distributed with mean $\lambda$. Upon using transform methods similar to those used to derive Eq. 8, we obtain the following factorial moments (see Appendix 1 for more details):

29 $$\begin{aligned} X^{(1)}(a;t)&= {\left\{ \begin{array}{ll} \lambda e^{-\lambda (a-t)}, &{} t<a\\ \beta e^{\beta (t-a)}, &{} t>a \end{array}\right. },\nonumber \\ X^{(2)}(a,b;t)&= {\left\{ \begin{array}{ll} 0, &{} t<a<b\\ \lambda \beta e^{-\lambda (b-a)}e^{(\lambda +\beta )(t-a)}, &{} a<t<b\\ 2\beta ^2 e^{-\beta (b-a)}e^{2\beta (t-a)}, &{} a<b<t \end{array}\right. }. \end{aligned}$$

We have given $X^{(2)}(a,b;t)$ for only $a<b$ since the region $a>b$ can be found by imposing symmetry of the age arguments in $X^{(2)}$. After using Eq. 22 to convert $X^{(1)}$ and $X^{(2)}$ into $Y^{(1)}$ and $Y^{(2)}$, we can use Eqs. 26 and 27 to find age-structured moments, particularly the mean and variance for the number of individuals that have age in the interval [*a*, *b*]:

30 $$\begin{aligned} \mathrm {E}(Y_{[a,b]}(t))&= {\left\{ \begin{array}{ll} e^{\lambda (t-a)}-e^{\lambda (t-b)}, &{} t<a<b\\ e^{\beta (t-a)}-e^{\lambda (t-b)}, &{} a<t<b\\ e^{\beta (t-a)}-e^{\beta (t-b)}, &{} a<b<t, \end{array}\right. }\end{aligned}$$

31 $$\begin{aligned} \mathrm {Var}(Y_{[a,b]}(t))&= {\left\{ \begin{array}{ll} e^{2\lambda t}(e^{-\lambda a}-e^{-\lambda b})(-e^{-\lambda a}+e^{-\lambda b}+e^{-\lambda t}), &{} t<a<b\\ (e^{\beta (t-a)}-e^{\lambda (t-b)}) (e^{\beta (t-a)} +e^{\lambda (t-b)}-1), &{} a<t<b\\ e^{2\beta t}(e^{-\beta a}-e^{-\beta b})(e^{-\beta a}-e^{-\beta b}+e^{-\beta t}), &{} a<b<t. \end{array}\right. } \end{aligned}$$

Note that in the limits $a \rightarrow 0$ and $b\rightarrow \infty$, we recover the expected exponential growth of the total population size $\mathrm {E}(Y_{[0,\infty ]}) = e^{\beta t}$ for a Yule-Furry process. We also recover the known total population variance $\mathrm {Var}(Y_{[0,\infty ]}) = e^{\beta t}(e^{\beta t}-1)$.

### Full Solution

Equation 17 defines a set of coupled linear integro-differential equations in terms of the density $\rho _n(\mathbf{a}_n;t)$. In [16], we derived explicit analytic expressions for $\rho _n(\mathbf{a}_n;t)$ in the limits of pure death and pure birth. Here, we outline the derivation of a formal expression for the full solution. To do so, it will prove useful to revert to the following representation for the density:

32 $$\begin{aligned} f_n(\mathbf{a}_n;t) \equiv n!\rho _n(\mathbf{a}_n;t). \end{aligned}$$

If $\mathbf{a}_n$ is restricted to the ordered region such that $a_1 \le a_2 \le \cdots \le a_n$, $f_n$ can be interpreted as the probability density for age-ordered individuals (see [16] for more details). We will consider $f_n$ as a distribution over $\mathbb {R}^n$; however, its total integral (*n*!) is not unity and it is not a probability density. We can use Eq. 32 to switch between the two representations, but simpler analytic expressions for solutions to Eq. 17 result when $f_n(\mathbf{a}_n;t)$ is used.

**Fig. 2:**
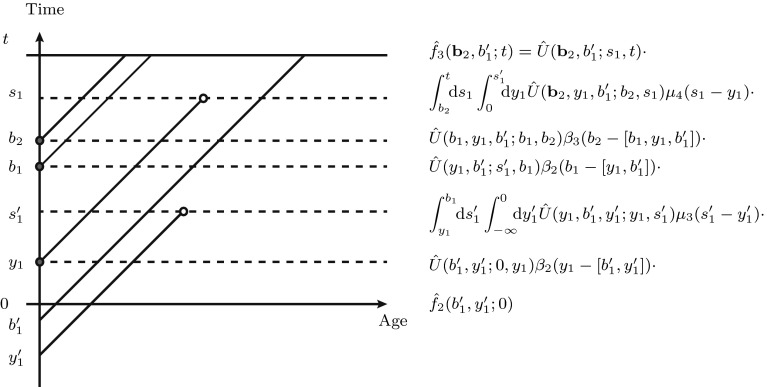
A sample birth death process over the time interval [0, *t*]. *Red* and *white circles* indicate births and deaths within [0, *t*]. The variables $b_i>0$ and $b'_j<0$ denote TOBs of individuals present at time *t*, while $y_i>0, y'_j<0$, and $s_i,s'_j \in [0,t]$ indicate birth and death times of individuals who have died by time *t*. Terms arising from application of the recursion in Eq. 34 and boundary condition of Eq. 18 are given to the right

To find general solutions for $f_n(\mathbf{a}_n;t)$ expressed in terms of an initial distribution, we replace $\rho _n(\mathbf{a}_n;t)$ with $f_n(\mathbf{a}_n;t)/n!$ in Eq. 17 and use the method of characteristics to find a solution. Examples of characteristics are the diagonal timelines portrayed in Fig. 2. So far, everything has been expressed in terms of the natural parameters of the system; the age $\mathbf{a}_{n}$ of the individuals at time *t*. However, $\mathbf{a}_{n}$ varies in time complicating the analytic expressions. If we index each characteristic by the time of birth (TOB) $b=t-a$ instead of age *a*, then *b* is fixed for any point (*a*, *t*) lying on a characteristic, resulting in further analytic simplicity. We use the following identity to interchange between TOB and age representations:

33 $$\begin{aligned} \hat{f}_n(\mathbf{b}_n;t) \equiv f_n(\mathbf{a}_n;t),\quad \mathbf{b}_n = t - \mathbf{a}_n. \end{aligned}$$

We will abuse notation throughout our derivation by identifying $t-\mathbf{a}_n\equiv [t-a_1,t-a_2,\ldots ,t-a_n]$. The method of characteristics then solves Eq. 17 to give a solution of the following form, for any $t_0 \ge \max \{\mathbf{b}_n\}$

34 $$\begin{aligned} \hat{f}_n(\mathbf{b}_n;t)= \hat{f}_n(\mathbf{b}_n;t_0)\hat{U}_n(\mathbf{b}_n;t_0,t) +\displaystyle \int _{t_0}^t \mathrm{d}s \int _{-\infty }^s \mathrm{d}y \hat{U}_n(\mathbf{b}_n;s,t) \hat{f}_{n+1}(\mathbf{b}_n,y;s)\mu _{n+1}(s-y). \end{aligned}$$

This equation is defined in terms of a propagator $\hat{U}_n(\mathbf{b}_m;t_0,t) \equiv U_n(\mathbf{a}_m;t_0,t)$ that represents the survival probability over the time interval $[t_0,t]$, for *m* individuals born at times $\mathbf{b}_m$, in a population of size *n*,

35 $$\begin{aligned} \hat{U}_n(\mathbf{b}_m;t_0,t)=\exp \left[ -\sum _{i=1}^{m} \int _{t_0}^t\gamma _{n}(s-b_i)\mathrm{d}s\right] , \end{aligned}$$

where we have again used the definition $\gamma _n(a)=\beta _n(a)+\mu _n(a)$. The propagator $\hat{U}$ satisfies certain translational properties:

36 $$\begin{aligned} \hat{U}_n(\mathbf{b}_m;t_0,t)&=\prod _{i=1}^m\hat{U}_n(b_i;t_0,t), \end{aligned}$$

37 $$\begin{aligned} \hat{U}_n(\mathbf{b}_m;t_0,t)&=\hat{U}_n(\mathbf{b}_m;t_0,t') \ \hat{U}_n(\mathbf{b}_m;t',t). \end{aligned}$$

The solution $\hat{f}_n$ applies to any region of phase space where $t_0\ge \max \{\mathbf{b}_n\}$. If $t_0=\max \{\mathbf{b}_n\}$, say $t_0=b_n$, then we must invoke the boundary conditions of Eq. 18 to replace $\hat{f}_n (\mathbf{b}_{n-1},b_n;b_n)$ with $\hat{f}_{n-1} (\mathbf{b}_{n-1};b_n)\beta _{n-1}(b_n-\mathbf{b}_{n-1})$, where we have and will henceforth use the notation

38 $$\begin{aligned} \beta _{n-1}(b_n-\mathbf{b}_{n-1})&\equiv \beta _{n-1}(b_n-[b_{1}, b_{2},\ldots ,b_{n-1}])\nonumber \\ \,&\equiv \sum _{i=1}^{n-1}\beta _{n-1}(b_n-b_i). \end{aligned}$$

Eq. 34 is then used to propagate $\hat{f}_{n-1} (\mathbf{b}_{n-1};b_n)$ backwards in time. To obtain a general solution, we need to repeatedly back-substitute Eq. 34 and the associated boundary condition, resulting in an infinite series of integrals. However, each term in the resultant sum can be represented by a realization of the birth-death process. We represent any such realization across time period [0, *t*], such as that given in Fig. 2, as follows.

Let $\mathbf{b}_m \in [0,t]$ and $\mathbf{b}'_n<0$ denote the TOBs for *m* individuals born in the time interval [0, *t*], and *n* founder individuals, all alive at time *t*. Next, define $\mathbf{y}_k \in [0,t]$ and $\mathbf{y}'_\ell <0$ to be the TOBs of *k* individuals born in the time interval [0, *t*] and $\ell$ founder individuals, respectively. Here, all $k+\ell$ individuals are assumed to die in the time window [0, *t*]. Their corresponding times of death are defined as $\mathbf{s}_k$ and $\mathbf{s}'_\ell$, respectively. Thus, there will be $n+\ell$ individuals alive initially at time $t=0$ and $m+n$ individuals alive at the end of the interval [0, *t*].

Next, consider the realization in Fig. 2, where we start with the two individuals at time 0 with TOBs $b'_1$ and $y'_1$. The individual with TOB $b'_1$ survives until time *t*, while the individual with TOB $y'_1$ dies at time $s'_1$. Within the time interval [0, *t*] there are three more births with TOBs $b_1$, $b_2$ and $y_1$, the last of which has a corresponding death time of $s_1$, resulting in three individuals in total that exist at time *t*.

To express the distribution $\hat{f}_3(\mathbf{b}_2,b'_1;t)$ in terms of the initial distribution $\hat{f}_2(b'_1,y'_1;0)$, conditional upon three birth and two death events ordered such that $0<y_1<s'_1<b_1<b_2<s_1<t$, we start with the distribution $\hat{f}_2(b'_1,y'_1;0)$. Just prior to the first birth time $y_1$, we have two individuals, so that $\hat{f}_3(\cdot ;y_1^{-})\equiv 0$ and Eq. 34 yields $\hat{f}_2(b'_1,y'_1;y_1^{-}) = \hat{f}_2(b'_1,y'_1;0) \hat{U}(b'_1,y'_1;0,y_1)$ (the death term does not contribute). To describe the birth at time $y_1$, we use the boundary condition of Eq. 18 to construct $\hat{f}_3(b'_1,y'_1,y_1;y_1)= \hat{f}_2(b'_1,y'_1;y_1^{-})\beta _2(y_1-[b'_1,y'_1])$.

Immediately after $y_1$ and before the next death occurs at time $s'_1$, three individuals exist and $\hat{f}_2(\cdot ;y_1^{+})\equiv 0$. Now, only the death term in Eq. 34 contributes and

39 $$\begin{aligned} \hat{f}_2(y_1,b'_1;b_1^{-}) = \int _{y_1}^{b_1}\!\!\mathrm{d}s'_1 \int _{-\infty }^0\!\!\mathrm{d}y'_1\, \hat{U}(y_1,b'_1,y'_1;y_1,s'_1) \mu _3(s'_1-y'_1) \hat{f}_3(y_1,b'_1,y'_1;s'_1). \end{aligned}$$

Continuing this counting, we find the product of terms displayed on the right-hand side of Fig. 2.

Next, we use the translational properties indicated in Eqs. 36 and 37 to combine the propagators associated with Fig. 2 into one term: ${\hat{U}}(y'_1;0,s'_1){\hat{U}}(b'_1;0,t) {\hat{U}}(y_1;y_1,s_1)\hat{U}(b_1;b_1,t){\hat{U}}(b_2;b_2,t)$. In other words, each birth-death pair (*y*, *s*) is propagated along the time interval it survives; from $\max \{y,0\}$ to $\min \{s,t\}$. For example, the individual with TOB $b'_1<0$ survives across the entire timespan [0, *t*], whereas the individual with TOB $y_1$ is born and dies at times $y_1$ and $s_1$. These two individuals are propagated by the terms $U(b'_1;0,t)$ and $U(y_1;y_1,s_1)$, respectively. Provided the order $0<y_1<s'_1<b_1<b_2<s_1<t$ is preserved and the values $b'_1,y'_1<0$ are negative, the form of the integral expressions in Fig. 2 are preserved.

**Fig. 3:**
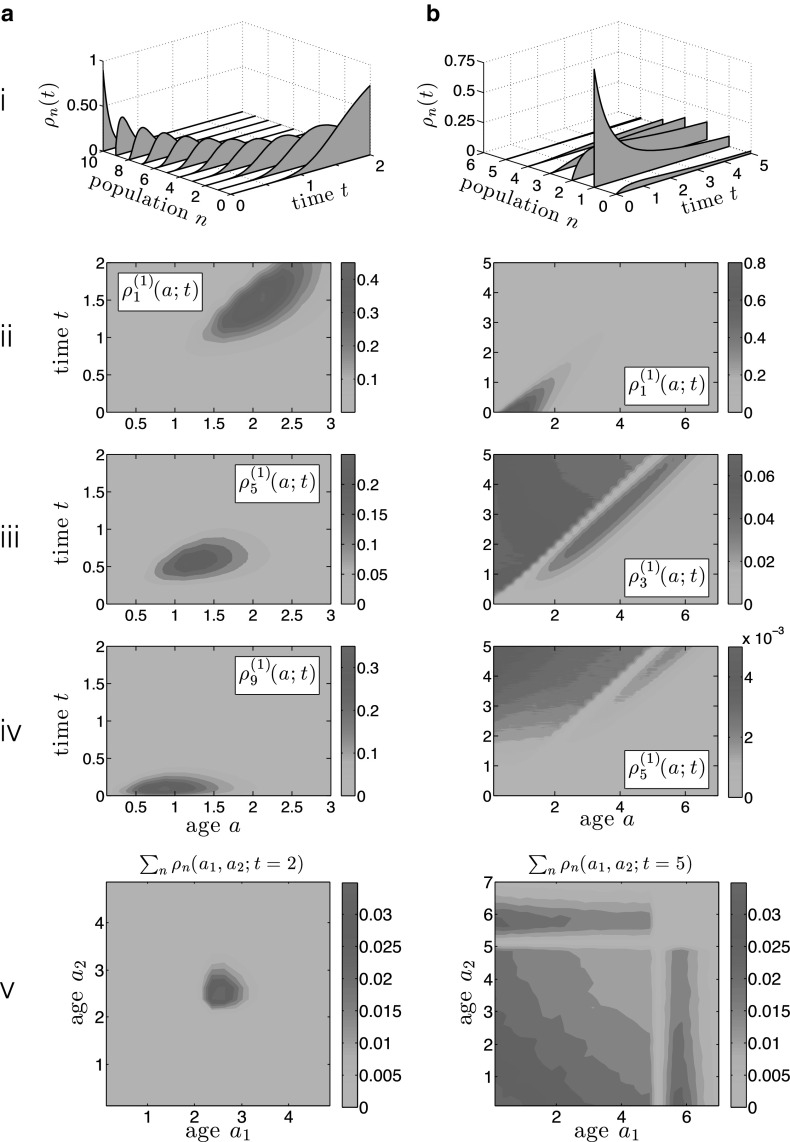
Monte–Carlo simulations of densities in age- and number-dependent birth-death processes. Column **a** shows results for a death-only process with a linear death rate function $\mu (a) = a$. We initiated all simulations from 10 individuals with initial age drawn from distribution $P(a) = 128a^{3}e^{-4a}/3$. In column **b**, we consider a budding-only birth process with a carrying capacity $K=5$ (in Eq. 10). Here, simulations were initiated with a single parent individual with an initial age also drawn from the distribution *P*(*a*). In (*i*), we plot the total number density $\rho _{n}^{(0)}(t)= \int \mathrm{d}\mathbf{a}\rho _n(\mathbf{a};t)$ for both processes. We also plot the single-particle density function $\rho _{n=1,5,9}(a;t=2)$ for the pure death process in **a**(*ii–iv*) and $\rho _{n=1,3,5}(a;t=5)$ for the limited budding process in **b**(*ii–iv*). Finally, the population-summed two-point correlations functions $\sum _{n}\rho _{n}^{(2)}(a_{1},a_{2};t)$ for pure death and pure budding are shown in panels **a**(*v*) and **b**(*v*)

After summing across all realizations $C_{m,k,\ell }$ (the configuration in Fig. 2 is one member of $C_{2,1,1}$) of the possible orderings of the birth and death times $\mathbf{b}_m$, $\mathbf{y}_k$, $\mathbf{y}'_\ell$, $\mathbf{s}_k$ and $\mathbf{s}'_\ell$, we can write the general solution to Eq. 34 in the form

40 $$\begin{aligned}&\hat{f}_{m+n}(\mathbf{b}_m,\mathbf{b}'_n;t) \nonumber \\&\quad = \sum _{k,\ell =0}^\infty \sum _{C_{m,k,\ell }} \int _{-\infty }^0\mathrm{d}\mathbf{y}'_\ell \cdot \int _{t^{-}(\mathbf{y}_k)}^{t^{+}(\mathbf{y}_k)}\mathrm{d}\mathbf{y}_k\cdot \int _{t^{-}(\mathbf{s}_k)}^{t^{+}(\mathbf{s}_k)}\mathrm{d}\mathbf{s}_k\cdot \int _{t^{-}(\mathbf{s}'_\ell )}^{t^{+}(\mathbf{s}'_\ell )}\mathrm{d}\mathbf{s}'_\ell \cdot \hat{f}_{n+l}(\mathbf{b}'_n,\mathbf{y}'_\ell ;0)\cdot \nonumber \\&\qquad \prod _{i=1}^m\hat{U}(b_i;b_i,t)\cdot \prod _{i=1}^k\hat{U}(y_i;y_i,s_i)\cdot \prod _{i=1}^n\hat{U}(b'_i;0,t)\cdot \prod _{i=1}^\ell \hat{U}(y'_i;0,s'_i)\cdot \prod _{i=1}^m\beta _{N(b_i)}(b_i-A(b_i))\cdot \nonumber \\&\qquad \prod _{i=1}^k \beta _{N(y_i)}(y_i-A(y_i))\cdot \prod _{i=1}^k \mu _{N(y_i)}(s_i-y_i)\cdot \prod _{i=1}^\ell \mu _{N(y'_i)}(s'_i-y'_i). \end{aligned}$$

The terms $t^{-}(\mathbf{x})$ and $t^{+}(\mathbf{x})$ refer to the times below and above $\mathbf{x}$ relative to the ordering of times $\mathbf{b}_m$, $\mathbf{y}_k$, $\mathbf{y}'_\ell$, $\mathbf{s}_k$ and $\mathbf{s}'_k$. For example, in Fig. 2, $t^{-}(\mathbf{b}_2)=[s'_1,b_1]$ and $t^{+}(\mathbf{b}_2)=[b_2,s_1]$ represent the lower and upper bounds of the vector $\mathbf{b}_2=[b_1,b_2]$ found from the ordering $0<y_1<s'_1<b_1<b_2<s_1$. The term *A*(*x*) represents the vector of TOBs of the individuals alive just prior to time *x*. The term *N*(*x*) represents the number of individuals alive just prior to time *x*.

Although analytic and complete, the solution given in Eq. 40 is unwieldy and difficult to implement. One can truncate the sum to remove low probability contributions, such as realizations containing improbable numbers of intermediary births and deaths, and perform numerical integration. However, this approach also rapidly becomes infeasible as the dimensions increase. Therefore, we explore the general solution via event-based Monte-Carlo simulation. We initialize the process with a number of samples obtained from an initial distribution. Each sample is represented by a vector $\mathbf{b}_n$ of birth times and is propagated forward in time. A timestep is chosen to be sufficiently small such that at most one birth or death event occurs within it, after which the vector $\mathbf{b}_n$ is updated. This process is continued until the required time has been reached. Although the high dimensionality makes it difficult to sample enough realizations to sufficiently explore the distribution $f_n(\mathbf{a}_n;t)$, lower dimensional marginal distributions such as $f_n^{(0)}(\cdot ;t)$, $f_n^{(1)}(a_1;t)$ and $f_n^{(2)}(a_1,a_2;t)$, and their counterparts $\rho _{n}$, can be sufficiently sampled.

Figure 3a, b show results from simulations of a pure death and a pure birth process, respectively. In Fig. 3a we assumed a population-independent linear death rate $\mu (a) = a$ and initiated the pure death process with 10 individuals with initial ages drawn from a gamma distribution with unit mean and standard deviation $\frac{1}{2}$. Fig. 3a(i) shows the simulated density which decreases in *n* with time. Figs. 3a(ii–iv) show that the weight of the reduced single-particle density function shifts to longer times and higher ages as the system size *n* is decreased. The sum over the population of the symmetric two-point correlation $\rho _{n}^{(2)}(a_{1}, a_{2};t=2)$ is shown in Fig. 3a(v). The observed structure indicates no correlations in the death only process and the peak at $a_{1}=a_{2} \approx 2.6$ reflects the fact that older individuals die faster, shifting the mean age slightly below the initial age plus the elapsed time (1 + 2 = 3). Fig. 3b shows results from Monte-Carlo simulations of a pure birth process with growth rate $\beta _{0} = 1$ and carrying capacity $K=5$ (Eq. 10). Here, we initiated the simulations with one individual with age drawn from the same gamma distribution $P(a) = 128 a^{3}e^{-4a}/3$. In this case, the reduced single-particle density exhibits peaks arising from both from the initial distribution and from birth (Fig. 3b(ii–iv)). The two-point correlation function $\sum _{n=0}^{\infty }\rho _{n}^{(2)}(a_{1}, a_{2};t=5)$ exhibits a similar multimodal structure as shown in (v). In all simulations at least 400,000 trajectories were aggregated and the results are in good agreement with analytic solutions to Eq. 17.

## Age-Structured Fission-Death Processes

We now derive a kinetic theory for a binary fission-death process, as depicted in Fig. 1b. We find a hierarchy of kinetic equations, analogous to Eqs. 17 and 18, and determine the mean behavior.

### Extended Liouville Equation for Fission-Death

The binary fission-death process is equivalent to a birth-death process except that parents are instantaneously replaced by *two* newborns. The process can also be thought of as a budding process in which the parent is instantaneously renewed. In order to describe both twinless individuals (singlets) and twins (a doublet), we have to double the dimensionality of our density functions. For example, in Fig. 1b at time $t_1$, we have two pairs of distinct twins, with four individuals having two ages, whereas at time $t_2$ we have two singlets and two doublets. Thus, we define the ages of current singlets and twins by $\mathbf{a}_m$ and $\mathbf{a}_n'$, respectively, where *m* is the number of singlets and *n* the number of pairs of twins. Transforming to the time-of-birth (TOB) representation, we define the TOB of current singlets and twins as $\mathbf{x}_m=t-\mathbf{a}_m$ and $\mathbf{y}_n=t-\mathbf{a}_n'$, respectively. For simplicity, we will assume that no simple birth processes occur and that particles grow in number only through fission. The function $\beta _{m,n}(a)$ is defined as the age-dependent fission rate of an individual (whether a singlet or a doublet) of age *a* when the system contains *m* singlets and *n* doublets. Similarly, we have death rate $\mu _{m,n}(a)$, and event rate $\gamma _{m,n}(a) = \beta _{m,n}(a)+ \mu _{m,n}(a)$. We suppose, for the moment, that the TOBs are ordered so that $x_1 \le x_2 \le \cdots \le x_m$ and $y_1 \le y_2 \le \cdots \le y_n$. The quantity $f_{m,n}(\mathbf{x}_{m};\mathbf{y}_{n})\mathrm{d}\mathbf{x}_m \mathrm{d}\mathbf{y}_n$ is then the probability of *m* singlets with ordered TOBs in $[\mathbf{x}_m,\mathbf{x}_m+\mathrm{d}\mathbf{x}_m]$ and *n* twin pairs with ordered TOBs in $[\mathbf{y}_n,\mathbf{y}_n+\mathrm{d}\mathbf{y}_n]$. The density $f_{m,n}$ satisfies the following equation:

41 $$\begin{aligned} \begin{array}{l} \displaystyle {\partial f_{m,n}(\mathbf{x}_{m};\mathbf{y}_{n};t)\over \partial t} +f_{m,n}(\mathbf{x}_{m};\mathbf{y}_{n};t)\left[ \sum _{i=1}^{m}\gamma _{m,n}(t-x_{i})+2\sum _{j=1}^{n}\gamma _{m,n}(t-y_{j})\right] = \\ \, \displaystyle \sum _{i=0}^{m}\int _{x_{i}}^{x_{i+1}} f_{m+1,n}(\mathbf{x}_{i},z,\mathbf{x}_{i+1,m};\mathbf{y}_{n};t)\mu _{m+1,n}(t-z)\mathrm{d}z \\ \, +\, \displaystyle 2\sum _{i=1}^{m}f_{m-1,n+1}(\mathbf{x}_m^{(-i)};\mathbf{y}_{i},x_{i}, \mathbf{y}_{i+1,n};t)\mu _{m-1,n+1}(t-x_{i}), \end{array} \end{aligned}$$

where the partial age vectors are defined as $\mathbf{x}_{i,j} = (x_i,\ldots ,x_j)$ and the singlet age vector, doublet age vector, and time arguments are separated by semicolons. The term $\mathbf{x}_m^{(-i)}=(x_1,\ldots ,x_{i-1},x_{i+1},\ldots ,x_m)$ represents the vector of all *m* singlet TOBs, except for the *i*th one. The first term on the right hand side of Eq. 41 represents the death of a singlet particle with an unknown TOB *z* in the interval $[x_i,x_{i+1}]$, while the second term describes the death of any one of two individuals in a pair of twins (with TOB $x_i$).

The associated boundary conditions are

42 $$\begin{aligned} f_{m,n}(\mathbf{x}_{m-1},t; \mathbf{y}_{n};t)&= 0,\end{aligned}$$

43 $$\begin{aligned} f_{m,n}(\mathbf{x}_{m}; \mathbf{y}_{n-1},t;t)&= 2\sum _{i=1}^{m} f_{m-1,n}(\mathbf{x}_m^{(-i)};\mathbf{y}_{n-1},x_{i};t)\beta _{m-1,n}(t-x_{i}) \nonumber \\&+\sum _{i=0}^{m} \int _{x_{i}}^{x_{i+1}}f_{m+1,n-1}(\mathbf{x}_{i},z,\mathbf{x}_{i+1,m}; \mathbf{y}_{n};t)\beta _{m+1,n-1}(t-z)\mathrm{d}z. \end{aligned}$$

The first term on the right-hand side above represents the fission of one of a pair of twins, generating a new pair of twins of age zero (TOB *t*), and leaving behind a singlet with TOB $x_{i}$. The second term represents the fission (and removal) of a singlet with unknown TOB *z*, giving rise to an additional pair of twins of age zero.

We now let $\mathbf{x}_m$ and $\mathbf{y}_n$ be unordered TOB vectors, and extend $f_{m,n}$ to the domain $\mathbb {R}^{m+n}$ by defining $f_{m,n}(\mathbf{x}_{m};\mathbf{y}_{n};t) = f_{m,n}(\mathcal{T}(\mathbf{x}_{m});\mathcal{T}(\mathbf{y}_{n});t)$, where $\mathcal {T}$ is the ordering operator. Note that $f_{m,n}$ is not a probability distribution under this extension; however, $\rho _{m,n}(\mathbf{x}_{m};\mathbf{y}_{n};t)\mathrm{d}\mathbf{x}_m \mathrm{d}\mathbf{y}_n = {1 \over m! n!}f_{m,n}(\mathbf{x}_{m};\mathbf{y}_{n};t)\mathrm{d}\mathbf{x}_m \mathrm{d}\mathbf{y}_n$ can be interpreted as the probability that we have a population of *m* singlets and *n* pairs of twins, such that if we randomly label the singlets $1,2,\ldots ,m$ and the doublets $1,2,\ldots ,n$, the $i^\mathrm {th}$ singlet has age in $[x_i,x_i+\mathrm{d}x_i]$ and the $j^\mathrm {th}$ doublet have age in $[x_j,x_j+\mathrm{d}x_j]$. The density $\rho _{m,n}$ obeys

44 $$\begin{aligned}&\frac{\partial \rho _{m,n}(\mathbf{x}_{m};\mathbf{y}_{n};t)}{\partial t} +\rho _{m,n}(\mathbf{x}_{m};\mathbf{y}_{n};t)\left[ \sum _{i=1}^{m}\gamma _{m,n}(t-x_{i})+2\sum _{j=1}^{n}\gamma _{m,n}(t-y_{j})\right] \nonumber \\&\quad = (m+1)\int _{-\infty }^{t} \rho _{m+1,n}(\mathbf{x}_{m},z;\mathbf{y}_{n};t)\mu _{m+1,n}(t-z)\mathrm{d}z \nonumber \\&\qquad +\, 2\left( {n+1\over m}\right) \sum _{i=1}^{m} \rho _{m-1,n+1}(\mathbf{x}_m^{(-i)};\mathbf{y}_{n},x_{i};t)\mu _{m-1,n+1}(t-x_{i}), \end{aligned}$$

with associated boundary condition

45 $$\begin{aligned} \rho _{m,n}(\mathbf{x}_{m-1},t; \mathbf{y}_n;t)&= 0,\nonumber \\ \rho _{m,n}(\mathbf{x}_{m}; \mathbf{y}_{n-1},t;t)&= {2\over m}\sum _{i=1}^{m}\rho _{m-1,n}(\mathbf{x}_m^{(-i)};\mathbf{y}_{n-1},x_{i};t) \beta _{m-1,n}(t-x_{i}) \nonumber \\&\quad + \left( {m+1\over n}\right) \int _{-\infty }^{t} \rho _{m+1,n-1}(\mathbf{x}_{m},z;\mathbf{y}_{n-1};t) \beta _{m+1,n-1}(t-z)\mathrm{d}z. \end{aligned}$$

Equations 44 and 45 provide a complete probabilistic description of the population of singlets and doublets undergoing fission and death.

### Mean-Field Behavior

Here, we analyze the mean-field behavior of the fission-death process by first integrating out unwanted variables from the full density $\rho _{m,n}(\mathbf{x}_m;\mathbf{y}_n;t)$ to construct marginal or “reduced” densities. Successive integrals over any number of the variables $\mathbf{x}_m$ and $\mathbf{y}_n$ can be performed, giving:

46 $$\begin{aligned} \rho _{m,n}^{(k,\ell )}(\mathbf{x}_{k};\mathbf{y}_{\ell };t) \equiv \int _{-\infty }^{t}\mathrm{d}\mathbf{x}'_{m-k}\int _{-\infty }^{t}\mathrm{d}\mathbf{y}'_{n-\ell } \rho _{m,n}(\mathbf{x}_k,\mathbf{x}'_{m-k};\mathbf{y}_{\ell },\mathbf{y}'_{n-\ell };t). \end{aligned}$$

For example, $\rho _{m,n}^{(0,0)}(;;t)$ is the probability of finding at time *t*, *m* singlets and *n* doublets, regardless of age. After integrating Eq. 44 we find the double hierarchy of equations

47 $$\begin{aligned}&\frac{\partial \rho _{m,n}^{(k,\ell )}(\mathbf{x}_{k};\mathbf{y}_{\ell };t)}{\partial t} +\rho _{m,n}^{(k,\ell )}(\mathbf{x}_{k};\mathbf{y}_{\ell };t) \left[ \sum _{i=1}^k\gamma _{m,n}(t-x_i)+ 2\sum _{i=1}^\ell \gamma _{m,n}(t-y_i) \right] \nonumber \\&\qquad + (m-k)\int _{-\infty }^t \rho _{m,n}^{(k+1,\ell )}(\mathbf{x}_{k},z;\mathbf{y}_{\ell };t) \gamma _{m,n}(t-z) \mathrm{d}z \nonumber \\&\qquad +2(n-\ell )\int _{-\infty }^t \rho _{m,n}^{(k,\ell +1)}(\mathbf{x}_{k};\mathbf{y}_{\ell },z;t) \gamma _{m,n}(t-z) \mathrm{d}z \nonumber \\&\quad = (m+1)\int _{-\infty }^t \rho _{m+1,n}^{(k+1,\ell )}(\mathbf{x}_{k},z;\mathbf{y}_{\ell };t) \mu _{m+1,n}(t-z) \mathrm{d}z \nonumber \\&\qquad + 2 \left( \frac{n+1}{m} \right) \sum _{i=1}^k \rho _{m-1,n+1}^{(k-1,\ell +1)}(\mathbf{x}_{k}^{(-i)};\mathbf{y}_{\ell },x_i;t) \mu _{m-1,n+1}(t-x_i) \nonumber \\&\qquad + 2 \left( \frac{n+1}{m} \right) (m-k)\int _{-\infty }^t \rho _{m-1,n+1}^{(k,\ell +1)}(\mathbf{x}_{k};\mathbf{y}_{\ell },z;t) \mu _{m-1,n+1}(t-z) \mathrm{d}z. \end{aligned}$$

Similarly, integrating Eq. 45 yields boundary conditions for the marginal densities:

48 $$\begin{aligned} \rho _{m,n}^{(k,\ell )}(\mathbf{x}_{k-1},t;\mathbf{y}_{\ell };t) = \,&\, 0, \nonumber \\ \rho _{m,n}^{(k,\ell )}(\mathbf{x}_k;\mathbf{y}_{\ell -1},t;t) =\,&\frac{2}{m}\sum _{i=1}^k \rho _{m-1,n}^{(k-1,\ell )}(\mathbf{x}_k^{(-i)};\mathbf{y}_{\ell -1},x_i;t) \beta _{m-1,n}(t-x_i) \nonumber \\&+ 2\left( \frac{m-k}{m}\right) \int _{-\infty }^t \rho _{m-1,n}^{(k,\ell )}(\mathbf{x}_k;\mathbf{y}_{\ell -1},z;t) \beta _{m-1,n}(t-z)\mathrm{d}z \nonumber \\&+ \left( \frac{m+1}{n}\right) \int _{-\infty }^t \rho _{m+1,n-1}^{(k+1,\ell -1)}(\mathbf{x}_k,z;\mathbf{y}_{\ell -1};t) \beta _{m+1,n-1}(t-z)\mathrm{d}z. \end{aligned}$$

We can now analyze the densities *X*(*x*, *t*) and *Y*(*y*, *t*), where $X(x,t)\mathrm{d}x$ is the probability that there exists at time *t* a singlet with TOB in $[x,x+\mathrm{d}x]$ and $Y(y,t) \mathrm{d}y$ is the probability that at time *t* we have one doublet with TOB in $[y,y+\mathrm{d}y]$. Analogous to Eq. 22, we define

49 $$\begin{aligned} X(x,t)&\equiv \sum _{m,n=0}^{\infty }m\rho _{m,n}^{(1,0)}(x;;t) = \sum _{m,n=0}^{\infty }m\int _{-\infty }^{t}\mathrm{d}\mathbf{x}_{m-1} \int _{-\infty }^{t}\mathrm{d}\mathbf{y}_{n}\rho _{m,n}(\mathbf{x}_{m-1},x;\mathbf{y}_{n};t), \nonumber \\ Y(y,t)&\equiv \sum _{m,n=0}^{\infty }n\rho _{m,n}^{(0,1)}(;y;t) = \sum _{m,n=0}^{\infty }n\int _{-\infty }^{t}\mathrm{d}\mathbf{x}_{m} \int _{-\infty }^{t}\mathrm{d}\mathbf{y}_{n-1} \rho _{m,n}(\mathbf{x}_{m};\mathbf{y}_{n-1},y;t). \end{aligned}$$

Upon setting $(k,\ell )=(1,0)$ and $(k,\ell )=(0,1)$, we multiply Eq. 47 by *m* and *n*, respectively, and sum both equations. If the fission and death rates $\beta _{m,n}(a)$ and $\mu _{m,n}(a)$ depend on population size, the resultant expressions are complex hierarchies which will be difficult to analyze. However, if $\beta _{m,n}(a)=\beta (a)$ and $\mu _{m,n}(a)=\mu (a)$ are size-independent, many cancellations occur and the resulting equations for *X* and *Y* simplify significantly, giving

50 $$\begin{aligned} \frac{\partial X}{\partial t} = (2Y-X)\gamma (t-x),\quad \frac{\partial Y}{\partial t} = -2Y\gamma (t-x). \end{aligned}$$

Similarly, repeating the operation on the boundary conditions in Eq. 48, we find boundary conditions for *X* and *Y*:

51 $$\begin{aligned} X(t,t) = 0,\quad Y(t,t) = \int _{-\infty }^{t}(X(z,t)+2Y(z,t)) \gamma (t-z)\mathrm{d}z \equiv B(t). \end{aligned}$$

Note that if $T=X+2Y$ is the total population density, Eqs. 50 and 51 reduce to McKendrick-von Foerster-like equations:

52 $$\begin{aligned} \frac{\partial T}{\partial t} = -\gamma (t-z)T,\quad T(t,t) = \int _{-\infty }^{t}T(z,t)\gamma (t-z)\mathrm{d}z. \end{aligned}$$

To solve Eqs. 50 and 51, we first define

53 $$\begin{aligned} U(x;t_1,t_2)=\exp \left[ -\int _{t_1}^{t_2}\gamma (s-x) ds\right] , \end{aligned}$$

and find solutions of the form

54 $$\begin{aligned} X(x,t)&= X(x,t_0)U(x;t_0,t)+2Y(x,t_0)U(x;t_0,t)(1-U(x;t_0,t)),\nonumber \\ Y(x,t)&= Y(x,t_0)U^{2}(x;t_0,t), \end{aligned}$$

provided $t_{0} \ge x$. For an initial time of $t=0$, we find, upon setting $t_0=\max \{0,x\}$,

55 $$\begin{aligned} X(x,t)={\left\{ \begin{array}{ll} 2B(x)U(x;x,t)(1-U(x;x,t)), &{} x>0,\\ X(x,0)U(x;0,t)+2Y(x,0)U(x;0,t)(1-U(x;0,t)), &{} x<0, \end{array}\right. } \end{aligned}$$

56 $$\begin{aligned} Y(x,t)={\left\{ \begin{array}{ll} B(x)U^{2}(x;x,t), &{} x>0,\\ Y(x,0)U^{2}(x;0,t), &{} x<0. \end{array}\right. } \end{aligned}$$

We now substitute Eqs. 55 and 56 into Eqs. 51 to find a Volterra equation for *B*(*t*):

57 $$\begin{aligned} B(t) = 2\int _0^{t}B(x)U(x;x,t) \beta (t-x) \mathrm{d}x + \int _{-\infty }^0[X(x,0)+2Y(x,0)]U(x;0,t)\beta (t-x) \mathrm{d}x. \end{aligned}$$

Equation 57 along with Eqs. 55 and 56 constitute a complete solution for the mean density of singlets and doublets. Eqs. 55 and 56 also show that the total population density, $T(x,t)= X(x,t)+2Y(x,t)$, takes on a simple form in terms of *B*(*t*):

58 $$\begin{aligned} T(x,t)={\left\{ \begin{array}{ll} 2B(t)U(x;x,t), &{} x>0,\\ T(x,0)U(x;0,t), &{} x<0, \end{array}\right. } \end{aligned}$$

while the total mean population $T(t)= \int _{0}^{\infty }T(x,t)\mathrm{d}x$ is given by

59 $$\begin{aligned} T(t) = 2\int _0^{t}B(x)U(x;x,t) \mathrm{d}x + \int _{-\infty }^0 T(x,0)U(x;0,t) \mathrm{d}x. \end{aligned}$$

Before analyzing a specific model of the fission-death process, we will first establish the equivalence of our noninteracting kinetic theory with the Bellman-Harris fission process (discussed in Sect. 2.3) in the mean-field limit.

### Mean-Field Equivalence to the Bellman-Harris Process

Consider a Bellman-Harris fission process with an inter-branching time distributed according to the function $g(\tau )$ and an associated cumulative density function defined by $G(t) = \int _{0}^{t}g(\tau )\mathrm{d}\tau$. Upon using the progeny distribution function $H(\cdot )$ given in Eq. 12, the Bellman-Harris model in Eq. 13 can be written equivalently as

60 $$\begin{aligned} F(z,t) = z(1-G(t)) + \int _0^t H(F(z,\tau )) g(t-\tau )\mathrm{d}\tau . \end{aligned}$$

If we restrict ourselves to a binary fission process, the progeny distribution function takes the form $H(y)=h_0+h_2y^2$, where $h_0$ and $h_2 = 1-h_{0}$ are the death and binary fission probabilities, conditional on an event taking place. Thus, the mean population defined as

61 $$\begin{aligned} T(t) \equiv \left. \frac{\partial F}{\partial z}\right| _{z=1} = \int _t^{\infty }g(\tau )\mathrm{d}\tau + 2h_2\int _0^{t}g(t-\tau )T(\tau )\mathrm{d}\tau \end{aligned}$$

has the Laplace-transformed solution

62 $$\begin{aligned} \tilde{T}(s) = \frac{1}{s}\frac{1-\tilde{g}(s)}{1-2 h_2\tilde{g}(s)}. \end{aligned}$$

We now show that the same result arises from our full noninteracting (population-independent $\beta (a)$ and $\mu (a)$) kinetic approach. Since the fission and death rates can be expressed as $\beta (y)=\frac{h_{2}g(y)}{1-G(y)}$ and $\mu (y)=\frac{h_{0} g(y)}{1-G(y)}$, Eq. 53 reduces to $U(x;x,t)=1-G(t-x)$ and $U(0;0,t)=1-G(t)$. Starting from a single individual with age zero, Eq. 59 can be written as

63 $$\begin{aligned} T(t) = 2\int _0^{t}B(x)(1-G(t-x)) \mathrm{d}x + (1-G(t)), \end{aligned}$$

which has the Laplace-transformed solution

64 $$\begin{aligned} \tilde{T}(s) = (2\tilde{B}(s)+1)\frac{1-\tilde{g}}{s}. \end{aligned}$$

Similarly, Eq. 57 becomes

65 $$\begin{aligned} B(t) = h_{2} g(t) + 2\int _0^{t}B(x)h_2 g(t-x) \mathrm{d}x, \end{aligned}$$

with Laplace-transformed solution

66 $$\begin{aligned} \tilde{B}(s) = \frac{h_2\tilde{g}(s)}{1-2 h_2\tilde{g}(s)}. \end{aligned}$$

Substituting Eq. 66 in Eq. 64 results in Eq. 62 for $\tilde{T}(s)$, explicitly establishing the mean-field equivalence between the Bellman-Harris approach and our kinetic theory. Note that in the Bellman-Harris formulation, the waiting-time distributions of either fission or death have the same distribution *g*(*a*). In our kinetic theory, these rates can have distinct distributions, $\beta _n(a)$ and $\mu _n(a)$, and can also depend on population size, providing much greater flexibility.

## A Fission-Only Model of Cell Division

We now consider explicit results for a simple fission-only model ($h_{2}=1$) of cell division where cell cycle times are rescaled to be $\Gamma$-distributed with unit mean and variance $\frac{1}{\alpha }$. This $\Gamma$-distribution and its Laplace transform $\tilde{g}(s)$ are explicitly

67 $$\begin{aligned} g(t) = \frac{\alpha ^\alpha }{\Gamma (\alpha )}t^{\alpha -1}e^{-\alpha t},\quad \tilde{g}(s) = \left( \frac{\alpha }{\alpha +s}\right) ^\alpha . \end{aligned}$$

Equation 66 for *B*(*t*) can then be solved to yield

68 $$\begin{aligned} B(t) = \mathcal {L}^{-1}_t\left( \frac{\alpha ^\alpha }{(s+\alpha )^\alpha -2\alpha ^\alpha }\right) =\alpha e^{-\alpha t}\mathcal {L}^{-1}_{(\alpha t)}\left( \frac{1}{s^\alpha -2}\right) . \end{aligned}$$

The inverse Laplace transform is detailed in Appendix 2 and involves contour integration that yields

69 $$\begin{aligned} B(t)= & {} -\frac{\alpha }{\pi }\int _0^{\infty }\frac{e^{-\alpha t(r+1)} r^\alpha \sin (\pi \alpha )}{r^{2\alpha }-4r^\alpha \cos (\pi \alpha )+4}\mathrm{d}r \nonumber \\&+\sum _{n=-\left\lfloor \frac{\alpha }{2}\right\rfloor }^{\left\lfloor \frac{\alpha }{2}\right\rfloor } 2^{\frac{1}{\alpha }-1}e^{{(2^{\frac{1}{\alpha }}\cos (\frac{2n\pi }{\alpha })-1)}\alpha t} \cos \left( 2^{\frac{1}{\alpha }}\alpha t\sin \left( \frac{2n\pi }{\alpha }\right) +\frac{2n\pi }{\alpha }\right) . \end{aligned}$$

Similarly, from Eq. 62 we have

70 $$\begin{aligned} T(t) = \mathcal {L}_t^{-1}\left( {\frac{1}{s}\frac{(s+\alpha )^\alpha -\alpha ^\alpha }{(s+\alpha )^\alpha -2\alpha ^\alpha }}\right) =e^{-\alpha t}\mathcal {L}_{(\alpha t)}^{-1} \left( {\frac{1}{s-1}\frac{s^\alpha -1}{s^\alpha -2}}\right) , \end{aligned}$$

which can also be evaluated via a similar Bromwich integral:

71 $$\begin{aligned} T(t)&= \displaystyle \frac{1}{\pi }\int _0^{\infty }\frac{e^{-\alpha t(r+1)}}{r+1} \frac{r^\alpha \sin (\pi \alpha )}{r^{2\alpha }-4r^\alpha \cos (\pi \alpha )+4}\mathrm{d}r \nonumber \\&\quad \, +\sum _{n=-\left\lfloor \frac{\alpha }{2}\right\rfloor }^{\left\lfloor \frac{\alpha }{2}\right\rfloor } \frac{2^{\frac{1}{\alpha }}}{2\alpha }e^{{(2^{\frac{1}{a}}\cos (\frac{2n\pi }{\alpha })-1)}\alpha t} \frac{2^{\frac{1}{\alpha }}\cos (2^{\frac{1}{\alpha }} \sin (\frac{2n\pi }{\alpha })\alpha t)\!-\!\cos (2^{\frac{1}{\alpha }} \sin (\frac{2n\pi }{\alpha })\alpha t\!+\!\frac{2n\pi }{\alpha })}{2^{\frac{2}{\alpha }}-2^{1+\frac{1}{\alpha }}\cos (\frac{2n\pi }{\alpha })+1}. \end{aligned}$$

**Fig. 4:**
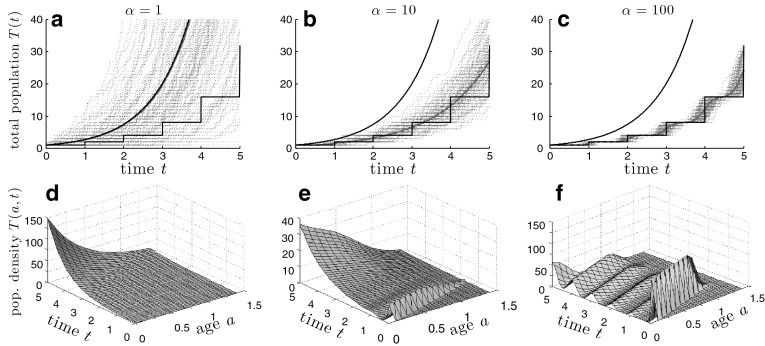
Plots of simulations and analytic results of a fission-only process with $\Gamma$-distributed branching times. **a**, **b**, and **c** show mean populations as a function of time for dispersion values $\alpha =1$, $\alpha =10$, and $\alpha = 100$, respectively. *Red dotted* trajectories are realizations of simulations, while the *solid red line* is the mean. The *blue dashed curve* is the mean population *T*(*t*) computed from Eq. 71 and is nearly indistinguishable from the *red solid curve*. The *upper* and *lower black lines* correspond to the continuous-time Markovian fission process and the discrete-time Galton-Watson process, respectively. **d**, **e**, and **f** depict the corresponding mean age-distributions *T*(*x*, *t*) computed from Eq. 58 but plotted as functions of time *t* and age *a*

For $\alpha = 1$, $g(t) = e^{-t}$ is exponentially distributed, and we find the simple growth law $T(t) = e^{t}$, which is equivalent to the result $\mathrm {E}(Y_{[0,\infty ]}) = e^{\beta t}$ found earlier in Sect. 3.1. This corresponds to a continuously compounded population. On the other hand, when $\alpha$ is increased, the $\Gamma$-distribution sharpens about unity. Figs. 4a–c show that as $\alpha$ increases, the mean population size *T*(*t*) tends towards that given by the discrete-time Galton–Watson step process, as would be expected. In the $\alpha \rightarrow \infty$ limit, the population compounds at discrete, evenly timed intervals leading to an overall lower population compared to that of a process with more frequent branching (smaller $\alpha$). In Figs. 4d–f, we have used the expression for *B*(*t*) in Eqs. 58 and 69 to give the mean age-time distribution *T*(*a*, *t*). Note that unlike the solution to the Bellman-Harris equation shown in Figs. 4a–c, the mean density *T*(*a*, *t*) (Eq. 58) resolves age structure.

## Spatial Models

We now illustrate how our age-structured kinetic model can be generalized to include spatial motion such as diffusion and convection. We will follow the approaches described in Webb [49] for incorporating spatial effects in age-structured simple birth-death processes. Since these methods are adaptations of the McKendrick-von Foerster equation, they are deterministic and ignore stochastic fluctuations in population size. In a manner similar to how the McKendrick-von Foerster equation was extended to the stochastic domain using Eq. 17, here, we outline how to generalize the age-structured spatial process discussed in [49] to incorporate stochasticity.

Consider a simple budding-mode birth-death process such that $\hat{\rho }_n(\mathbf{b}_n;\mathbf{q}_n;t)$ is the probability density for a population containing *n* randomly labelled individuals with TOBs $\mathbf{b}_n$*and* positions $\mathbf{q}_n$. Although $\hat{\rho }_n(\mathbf{b}_n;\mathbf{q}_n;t)$ is again invariant under permutations of variables associated with different individuals, the relative orders of $\mathbf{b}_n$ and $\mathbf{q}_n$ must be preserved. For example, $\hat{\rho }_2(b_1,b_2;q_1,q_2;t) = \hat{\rho }_2 (b_2,b_1;q_2,q_1;t)$. For ease of presentation, we assume a one-dimensional system; generalizations to higher spatial dimensions are straightforward. We further suppose that individuals are undergoing identical, independent diffusion processes with diffusion constant *D*. Examples of other spatial processes that may be combined with stochastic age-structured kinetics can be found in [49]. We suppose that $\beta _n(a;q)$ and $\mu _n(a;q)$ are birth and death rates for an individual with age *a* and at spatial position *q* in a population of size *n*. Finally, the initial position of each newborn is determined by the position of the parent at the time of birth. The extended theory is described by the following kinetic equation for $\hat{\rho }_n(\mathbf{b}_n;\mathbf{q}_n;t)$:

72 $$\begin{aligned} \frac{\partial \hat{\rho }_{n}(\mathbf{b}_n;\mathbf{q}_n;t)}{\partial t} =&-\hat{\rho }_{n}(\mathbf{b}_n;\mathbf{q}_n;t) \sum _{i=1}^n\gamma _{n}(t-b_i,q_i)+ D\sum _{i=1}^n \frac{\partial ^2}{\partial q_i^2}\hat{\rho }_n(\mathbf{b}_n;\mathbf{q}_n;t) \nonumber \\&+(n+1)\int _{-\infty }^{t}\!\!\mathrm{d}y \int _{\mathbb {R}} \mathrm{d}q' \hat{\rho }_{n+1}(\mathbf{b}_n,y;\mathbf{q}_n,q';t)\mu _{n+1}(t-y,z). \end{aligned}$$

The corresponding boundary condition capturing the influx of newborn individuals is

73 $$\begin{aligned} \rho _n(\mathbf{b}_ {n-1},t;\mathbf{q}_n;t) = \frac{1}{n}\sum _{i=1}^{n-1}\rho _{n-1}(\mathbf{b}_{n-1};\mathbf{q}_{n-1};t)\beta (t-b_i,q_i)\delta (q_n-q_i), \end{aligned}$$

which differs slightly from that in Eq. 18. In the original formulation, we do not track which individual is the parent of a newborn, whereas here the newborn has the same position ($q_n$) as the parent ($q_i$), setting its identity as the $i^\mathrm {th}$ individual. In addition to a boundary condition, Eq. 72 requires an initial condition $\rho _n(\mathbf{b}_n;\mathbf{q}_n;0)$ to specify both the initial TOB and initial position of individuals.

As with our earlier analyses, we first express $\rho _n$ in terms of $\rho _{n+1}$ by introducing the propagator $U_n(\mathbf{b}_n;\mathbf{q}_{n};t_0,t)=\exp \left[ -\sum _{i=1}^n\int _{t_0}^t \gamma _n(s-b_i,q_i)\mathrm{d}s\right]$, which enables us to transform Eq. 72 to an inhomogeneous heat equation for the function $U_n^{-1}\rho _n$,

74 $$\begin{aligned}&\frac{\partial }{\partial t}\left[ U_n^{-1}(\mathbf{b}_n;\mathbf{q}_{n};t_0,t)\rho _n\right] \nonumber \\&\quad = D\sum _{j=1}^n\frac{\partial ^2}{\partial q_j^2}\left[ U_n^{-1}\rho _n\right] +\, (n\!+\!1)U_n^{-1}\int _{-\infty }^{t} \mathrm{d}y \int _\mathbb {R} \! \mathrm{d}z \rho _{n+1}(\mathbf{b}_n,y;\mathbf{q}_n,z;t)\mu _{n+1}(t-y,z),\nonumber \\ \end{aligned}$$

whose solution can be expressed in the form [5]

75 $$\begin{aligned} \rho _n(\mathbf{b}_n;\mathbf{q}_n;t) =\, \,&U_n(\mathbf{b}_n;\mathbf{q}_{n};t_0,t)\int _{{\mathbb R}^n}\!\!\mathrm{d}\mathbf{q}'_n N_{\mathbf{q}_n}(\mathbf{q}'_n,2D(t-t_0)I_n)\rho _n(\mathbf{b}_n;\mathbf{q}'_n;t_0) \nonumber \\&+(n+1)\int _{t_0}^t \mathrm{d}s U_n(\mathbf{b}_n;\mathbf{q}_{n};s,t)\int _{{\mathbb R}^m} \!\!\mathrm{d}\mathbf{q}'_n N_{\mathbf{q}_n}(\mathbf{q}'_n,2D((t-t_0)-s)I_n) \nonumber \\&\times \int _{-\infty }^s \!\!\mathrm{d}y \int _{\mathbb {R}} \mathrm{d}z \,\rho _{n+1}(\mathbf{b}_n,y;\mathbf{q}'_n;z;s)\mu _{n+1}(s-y,z). \end{aligned}$$

Here, $I_n$ denotes the $n\times n$ identity matrix and $N_{\mathbf{q}}(\mathbf{x},\Sigma )$ is the multivariate normal density for the vector $\mathbf{q}$ arising from a distribution with mean $\mathbf{x}$ and covariance $\Sigma$. This result expresses $\rho _{n}$ in terms of $\rho _{n+1}$ and is analogous to Eq. 34. This solution is valid provided $t_0>\max \{\mathbf{x}\}$; for $t_0=\max \{\mathbf{x}\}$, we must invoke the boundary condition. One can then use Eq. 75 and the boundary condition to search for explicit solutions in much the same way as we did for our spatially independent kinetic theory.

## Summary and Conclusions

We have developed a complete kinetic theory for age-structured birth-death and fission-death processes that allow for systematic and and self-consistent incorporation of interactions at the population level. Our overall result in [16], which we extend here, is the derivation of a kinetic theory for stochastic age-structured populations. The kinetic equations can be written in terms of a BBGKY-like hierarchy (or a double hierarchy in the case of fission). Methods of approximation and closure typically employed in gas/liquid kinetic theory, plasma physics, or fluid dynamics can then be applied.

The analysis presented in this paper provides three new results. First, in Eq. 24, we have shown that the factorial moments of the age structure can be described by an equation that naturally generalizes the McKendrick-von Foerster equation. In particular, for population-independent birth, death, and fission rates we can determine the variance of the population size for specific age groups in a population, something that was not previously feasible without some form of approximation.

Second, in Eqs. 17 and 18, we develop a complete probabilistic description of a population undergoing a binary fission and death process. Although a general analytic solution to these systems can be written down (Eq. 40), it is difficult to calculate and further work is needed to identify analytic techniques or numerical schemes that can more readily provide solutions. The methods we have introduced can also be viewed as a continuum limit of matrix population models.

Third, we also outlined how to incorporate spatial dependence of birth and death into our age-structured kinetic theory. We considered only the simplest model of free diffusion in which individuals to not interact spatially. Spatially-mediated interactions can be incorporated by way of a “collision operator” in a full theory that treats both age and space kinetically.

Finally, we note that the overall structure of our model is semi-Markov. That is, birth, death, and fission rates depend on only the time since birth of an individual and not on, for example, the number of generations removed from a founder. Such lineage aging processes are often important in cell biology e.g., the Hayflick limit [21]) and would require extension of our state space to include generational class [52]. These extensions will be explored in future work.

## Figures and Tables

**Fig. 5:**
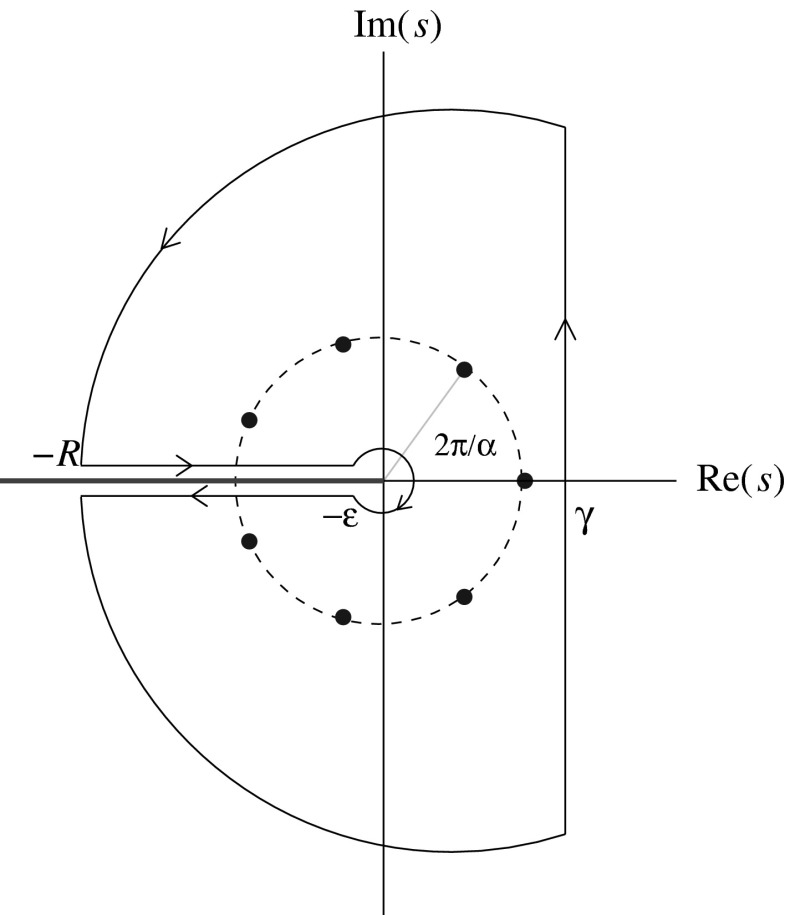
Bromwich integral for calculating the inverse Laplace transform in Eq. 80. The integral along $\gamma$ is evaluated using the residues at the poles and the integrals along the branch cut in Cauchy’s theorem

## Appendix 1: Second Factorial Moment Derivation

We outline how to derive Eq. 29. Assume the initial population is described by $X^{(1)}(a;0)=\lambda e^{-\lambda a}$ and $X^{(2)}(a,b;0) = 0$. Note that $X^{(1)}$ is just the solution to the McKendrick-von Foerster equation given by the expression in Eq. 5. We can determine $X^{(2)}$ via Eq. 28 if we are able to identify the boundary condition $B(a,t) \equiv X^{(2)}(a,0;t) \equiv X^{(2)}(0,a;t)$. After setting $m=\min \{a,b,t\}$ in Eq. 28, we substitute the expressions for $X^{(2)}$ into the boundary condition Eq. 25 to give the following equation for *B*(*a*, *t*):

76 $$\begin{aligned} B(a,t) = \frac{\beta }{2}X^{(1)}(a;t)+ \beta {\left\{ \begin{array}{ll} \int _0^tB(a-b,t-b) \mathrm{d}b, &{} t<a,\\ \int _0^aB(a-b,t-b)\mathrm{d}b +\int _a^\infty B(b-a,t-a)\mathrm{d}b, &{} t>a. \end{array}\right. } \end{aligned}$$

An expression for *B*(*a*, *t*) in the region $t<a$ can be obtained by solving along characteristics such as those portrayed in Fig. 2. We first define $C(\alpha ,\tau )=B(a,t)$, where $\alpha =a-t, \tau = t$, so that

77 $$\begin{aligned} C(\alpha ,\tau ) = \frac{\beta }{2}X^{(1)}(\alpha +\tau ;\tau )+ \beta \int _0^t C(\alpha ,\tau -b)\mathrm{d}b. \end{aligned}$$

A Laplace transform with respect to $\tau$ can then be used to find $B(a,t) = \frac{\beta \lambda }{2} e^{-\lambda a}e^{(\lambda +\beta )t}$.

For $t>a$, note that the second integral in Eq. 76 extends into the region $t<a$, for which we now have an expression. Upon separating the integral into two parts, and similarly defining $C(\alpha ,\tau )=B(a,t)$, where $\alpha =a, \tau = t-a$ along characteristics, we find

78 $$\begin{aligned} C(\alpha ,\tau ) = \frac{\beta ^2}{2}e^{\beta \tau } +\beta \int _0^\alpha C(b,\tau )\mathrm{d}b +\beta \int _0^\tau C(b,\tau -b)\mathrm{d}b +\frac{\beta \lambda }{2}\int _{\tau +\alpha }^\infty e^{-\lambda (b-\alpha )}e^{(\lambda +\beta )\tau }\mathrm{d}b. \end{aligned}$$

A double Laplace transform in variables $\alpha$ and $\tau$ results in:

79 $$\begin{aligned} \hat{C}(u,v) = \frac{\beta }{u}\left( \hat{C}(u,v) +\hat{C}(v,v)\right) +\frac{\beta ^2}{u}\frac{1}{v-\beta }, \end{aligned}$$

from which we find $\hat{C}(v,v) = \frac{\beta ^2}{(v-\beta )(v-2\beta )}$ and so $\hat{C}(u,v) = \frac{\beta ^2}{(u-\beta )(v-2\beta )}$. A double Laplace inversion then gives $B(a,t) = \beta ^2 e^{-\beta a}e^{2\beta t}$, from which $X^{(2)}$ can be uniquely determined from Eq. 28.

## Appendix 2: Bromwich Integral Calculation

Since the inverse Laplace transform provided by the Bromwich integral

80 $$\begin{aligned} \mathcal {L}^{-1}_{t}\left( \frac{1}{s^\alpha -2}\right) =\frac{1}{2\pi i} \int _{\gamma -i\infty }^{\gamma +i\infty }\frac{e^{st}}{s^\alpha -2}\mathrm{d}s \end{aligned}$$

involves a branch point at $s=0$, we construct a branch cut along the negative real axis and define $s=re^{i\theta }$ where $\theta \in (-\pi ,\pi )$. The denominator $s^{\alpha }-2$ also produces poles at $s=2^{\frac{1}{\alpha }}e^{i\frac{2n\pi }{\alpha }}$ where *n* is an integer with $|n| \le \left\lfloor {\frac{\alpha }{2}}\right\rfloor$. The contour required for the Bromwich integral is shown in Fig. 5 and is evaluated using Cauchy’s residue theorem.

The integrals around the outer perimeter and the origin contribute zero in the limit as $R \rightarrow \infty$ and $\varepsilon \rightarrow 0$. The branch cuts and poles provide the nonzero contributions. First, consider the integrals along the branch cut. Writing the variable *s* as $re^{i\theta }$, for $\theta = \pm \pi$, we integrate $\frac{1}{2\pi i}\frac{e^{st}}{s^{\alpha }-2}$ along the two sides to give

81 $$\begin{aligned} \frac{1}{2\pi i}\int _\infty ^0\frac{e^{-rt}(\mathrm{d}r e^{i\pi })}{r^\alpha e^{i\pi \alpha }-2} +\frac{1}{2\pi i}\int _0^\infty \frac{e^{-rt}(\mathrm{d}r e^{-i\pi })}{r^\alpha e^{-i\pi \alpha }-2} = -\frac{1}{\pi }\int _0^\infty \frac{e^{-rt}r^\alpha \sin (\pi \alpha )\mathrm{d}r}{r^{2\alpha }-4r^\alpha \cos (\pi \alpha )+4}. \end{aligned}$$

Next, we need to consider the poles at positions $s=2^\frac{1}{\alpha }e^\frac{2n\pi i}{\alpha }$ for $|n| \le \lfloor {\frac{\alpha }{2}}\rfloor$. L’Hôpital’s rule leads to

82 $$\begin{aligned} \lim _{s \rightarrow 2^\frac{1}{\alpha }e^\frac{2n\pi i}{\alpha }} \left\{ \frac{s-2^\frac{1}{\alpha }e^\frac{2n\pi i}{\alpha }}{s^\alpha -2}\right\} = \lim _{s \rightarrow 2^\frac{1}{\alpha }e^\frac{2n\pi i}{\alpha }}\left\{ \frac{1}{\alpha s^{\alpha -1}}\right\} = \alpha ^{-1}2^{\frac{1}{\alpha }-1}e^{\frac{2n\pi i}{\alpha }}. \end{aligned}$$

If $r_n$ is the residue for the function $\frac{e^{st}}{s^\alpha -2}$ at the pole $s=2^\frac{1}{\alpha }e^\frac{2n\pi i}{\alpha }$, we can write

83 $$\begin{aligned} r_n+r_{-n} = 2\mathrm {Re}\left\{ \alpha ^{-1}2^{\frac{1}{\alpha }-1}e^{\frac{2n\pi i}{\alpha }}e^{2^{\frac{1}{\alpha }}e^{\frac{2n\pi i}{\alpha }}t}\right\} = \frac{2^{\frac{1}{\alpha }}}{\alpha }e^{2^{\frac{1}{\alpha }}\cos \left( \frac{2n\pi }{\alpha }t\right) } \cos \left( 2^{\frac{1}{\alpha }}\sin \left( \frac{2n\pi }{\alpha }\right) +\frac{2n\pi }{\alpha }\right) .\nonumber \\ \end{aligned}$$

Combining the contributions from the branch cut and the residues results in $\mathcal {L}_{(t)}^{-1}\left( \frac{1}{s^\alpha -2}\right)$, which, when substituted into Eq. 68, gives the final result in Eq. 69.

The derivation for the Laplace inversion in Eq. 70 is similar. Note that the value $s=1$ is a removable singularity and the same set of poles and integration paths around branch cuts apply. Details are left to the reader.
